# Engineering the Catalytic Sites and Porous Framework of Biomass-Derived Carbon Aerogels for Zinc–Air Batteries and Pressure Sensors: A Review

**DOI:** 10.3390/gels12100917

**Published:** 2026-10-13

**Authors:** Chenyue Yu, Yanglei Chen, Lei Zhang, Ru Li, Lucian A. Lucia, Xiaofei Zhou, Guihua Yang

**Affiliations:** 1 Key Laboratory of Pulp and Paper Science & Technology of Ministry of Education, State Key Laboratory of Green Papermaking and Resource Recycling, Qilu University of Technology ( Shandong Academy of Sciences), Jinan 250353, China; ycy419323@163.com (C.Y.); ygh@qlu.edu.cn (G.Y.); 2 Shandong Sport University, Jinan 250102, China; 3 Department of Forest Biomaterials, North Carolina State University, Raleigh, NC 27695-8005, USA; lalucia@ncsu.edu

**Keywords:** bio-based carbon aerogels, pore regulation, heteroatom doping, zinc–air batteries, flexible sensors

## Abstract

The ever-growing global demand for sustainable energy has promoted the development of zinc–air batteries with high energy density. However, the slow multi-electron kinetic process of the oxygen reduction reaction and oxygen evolution reaction still restricts the practical commercialization of this battery technology. Biomass-based carbon aerogels can be constructed into tunable three-dimensional hierarchical structural frameworks, which provide high specific surface area and tunable structural characteristics, offering opportunities to improve mass transport and catalytic-site accessibility. This paper systematically reviews the latest research progress in the preparation and structural regulation of biomass-based carbon aerogels and elaborates on the determining effect of directional sol–gel processes, advanced drying techniques, and activation mechanisms on pore structure. To further improve the bifunctional electrocatalytic activity, this paper highlights the key doping and modification strategies, including nitrogen doping, multi-element co-doping, as well as the precise immobilization of non-noble metal nanoparticles and single-atom active sites. In addition to serving as high-efficiency, self-supporting, and flexible air cathodes for energy storage devices, the excellent mechanical rebound performance and continuous conductive network of this type of carbon aerogel also make it suitable for high-sensitivity pressure sensors. In conclusion, rational design and modification of biomass precursors can open up a green and sustainable development path for next-generation multifunctional flexible electronic devices.

## 1. Introduction

Due to the non-renewability of fossil energy and the resulting environmental pollution problems, the global demand for green and sustainable energy has been showing a continuously increasing trend. Hence, research and development of biomass-based energy materials based on renewable resources has become a focus research field. Among various electrochemical storage technologies, zinc–air batteries have shown excellent potential application prospects due to their exceptionally high theoretical energy density of 1086 Wh kg^−1^, low production cost, intrinsic safety, and excellent environmental compatibility. At the same time, their practical commercialization is still hindered by several critical challenges [1]. As the core reaction site of the battery, the air electrode suffers from intrinsically sluggish kinetics of the oxygen reduction reaction (ORR) and oxygen evolution reaction (OER), both of which involve multi-electron transfer pathways. In addition, the limited reaction region at the gas–liquid-solid three-phase interface restricts the efficiency of mass transport, while conventional carbon-based catalysts often exhibit low active-site density and poor cycling durability due to factors such as carbon corrosion and active-component detachment during operation [2]. Furthermore, the zinc anode in alkaline electrolytes is susceptible to hydrogen evolution corrosion and dendrite formation, which severely compromises both cycling life and safety. Consequently, the design and construction of highly active and durable air-electrode catalysts has become a key pathway for overcoming the performance bottlenecks of zinc–air batteries and accelerating their technological advancement and practical deployment [1].

Biomass offers broad availability, high renewability, and remarkable compositional diversity, including wood, cellulose, lignin, and numerous agricultural residues. Carbon aerogels prepared from bio-based materials as precursors have various advantages, including light weight, high porosity, and a special three-dimensional structure. Bio-based carbon aerogels have been investigated as air-electrode materials for zinc–air batteries. These natural precursors are intrinsically rich in carbon and possess complex but well-defined hierarchical architectures [3]. Benefiting from their inherent pores and framework, biomass precursors can be converted into carbon aerogels through sol–gel and freeze-drying processes, as well as carbonization or activation. Bio-based carbon aerogels possess a high specific surface area, adjustable pore structure, and abundant structural defects. These structural features provide a basis for regulating mass transport and the accessibility of catalytic sites in oxygen electrocatalysis [2]. Furthermore, various biomass sources may easily be combined with external nitrogen-rich additives or naturally contain heteroatoms such as O, N, S, and P, which enable in situ heteroatom doping during carbon formation, facilitating the construction of metal–nitrogen–carbon (M-N-C) coordination motifs, defect-rich active centers, and even stable single-atom anchoring sites [4]. Meanwhile, these features provide opportunities to regulate active-site exposure, electronic structure, and mass transport in air-electrode catalysts [5].

While numerous excellent reviews have comprehensively documented the general application of carbon nanomaterials in zinc–air batteries, most existing literature primarily focuses on traditional synthetic precursors. A critical gap remains in systematically bridging the targeted design of biomass-derived carbon aerogels with the emerging demands of flexible and wearable energy storage. This review advances beyond existing summaries by specifically addressing the unique structural and chemical complexities introduced by natural precursors (Table 1). Despite the well-documented sustainability of biomass aerogels, several critical unresolved issues continue to bottleneck their practical application, which serves as the primary motivation for this review. First, achieving precise, bottom-up control over hierarchical porosity remains profoundly challenging due to the intrinsic heterogeneity of complex precursors, such as fractionated lignin or industrial waste liquors, often leading to the burial of active sites within inaccessible micropores. Second, the electrocatalytic enhancement mechanisms in heteroatom-doped bio-aerogels are frequently treated as a black box; the literature urgently requires a systematic framework to decouple overlapping variables, such as topological defects, electronic modulation, and surface wettability. Finally, the intricate relationship between the cathode’s mass-transport architecture and total-cell failure mechanisms, including electrolyte dry-out and the consequent exacerbation of zinc anode dendrite proliferation, demands critical examination. By systematically addressing these specific unresolved issues, this review aims to provide a targeted roadmap for transitioning biomass-based carbon aerogels from fundamental material synthesis to practical, flexible device integration. In addition, bio-based carbon aerogels demonstrate enormous potential for application in pressure sensing. Their three-dimensional carbon frameworks, interconnected porous structures, and high specific surface areas provide excellent compressive resilience and mechanical stability [3]. These structural features are beneficial for oxygen electrocatalysis in zinc–air battery air cathodes and well suited for pressure sensing. Bio-based carbon aerogel sensors can convert small pressure changes into stable electrical signals while maintaining structural integrity and continuous conductivity [6]. Moreover, biomass precursors are abundant and structurally tunable, allowing the pore architecture and mechanical properties of carbon aerogels to be tailored to specific sensing requirements. It can be seen that bio-based carbon aerogels are regarded as multifunctional materials that combine practical performance with environmental sustainability.

**Table 1:** Comparison of related reviews with this work.

Review Article	Biomass-Derived Carbon Materials	Carbon Aerogel	Zn-Air Battery Applications	ORR/OER Active-Site Regulation	Structure-Performance Relationship	Pressure Sensing Applications	Energy Storage-Sensing Integration
[7]	✓	△	✓	✓	△	×	×
[8]	✓	△	✓	✓	△	×	×
[9]	✓	△	✓	△	△	×	×
[10]	✓	✓	△	△	✓	△	×
[11]	△	✓	△	△	✓	×	×
This work	✓	✓	✓	✓	✓	✓	✓

“✓” = extensively discussed; “△” = partially discussed; “×” = not covered.

**Figure 1:**
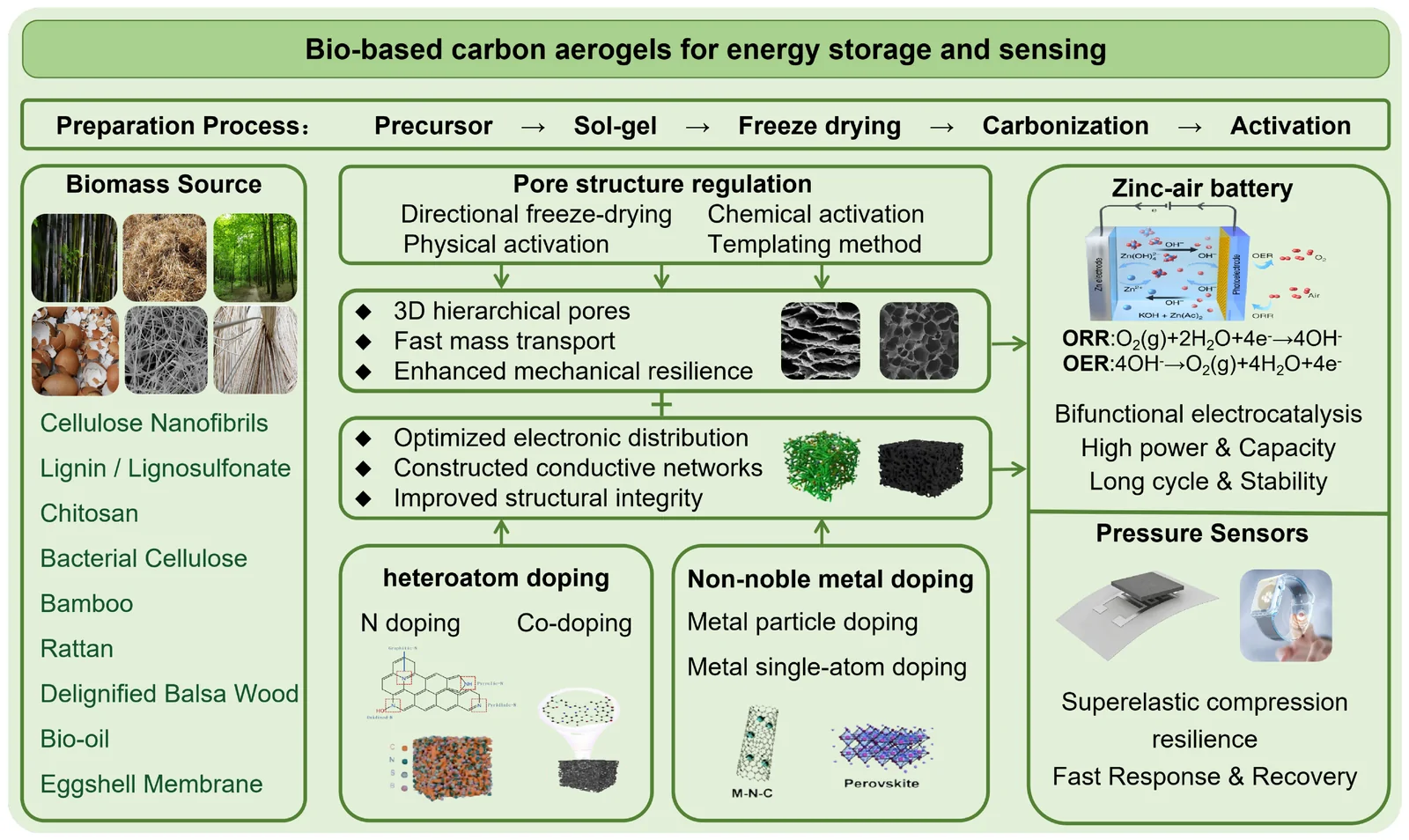
Summary diagram, including biomass sources, carbon aerogel preparation, doping, and applications.

Previous reviews mainly focused on biomass-derived carbon materials, porous carbon structures, or specific applications, whereas this review emphasizes the integrated relationship among precursor selection, structural regulation, active-site engineering, electrochemical performance, and pressure sensing applications. In this review, the recent advances in bio-based carbon aerogels used as air cathode materials for zinc–air batteries and their applications in pressure sensors are summarized and discussed. Figure 1 illustrates the overall research framework of bio-based carbon aerogels in this review. Carbon aerogels are prepared from biomass precursors such as cellulose nanofibers, lignin, bamboo, and eggshell membranes via sol–gel, freeze-drying, carbonization, and activation processes. Pore structure regulation, heteroatom doping, and non-precious metal doping are adopted for modification and performance optimization, so as to achieve ideal application performance. The main focuses include the drying methods of precursors, the pore structure regulation, the processes of doping with heteroatoms, and the construction of non-precious metal and single-atom active sites. The inherent structural advantages of biomass precursors are highlighted. The important roles of heteroatom doping and non-precious metal modification in enhancing bifunctional electrocatalytic performance for ORR and OER are further demonstrated by comparing the representative literature in this field. The dual potential of bio-based carbon aerogels in both energy storage and sensing applications is presented overall [12].

## 2. Preparation and Pore Structure Regulation of Bio-Based Carbon Aerogels

### 2.1. Preparation Methods and Structural Requirements of Carbon Aerogels

The first critical step in the preparation of biomass-based carbon aerogels is the sol–gel polymerization stage, which determines the initial network structure of the resulting carbon aerogel [13]. The evolution of this microstructure is highly sensitive to precursor composition and catalyst concentration. Specifically, increasing the catalyst-to-precursor ratio accelerates gel reaction kinetics, forming a skeleton with high cross-linking density and small pore size, which is highly suitable for charge storage. Conversely, reducing the catalyst ratio promotes the formation of a macroporous network that accelerates mass transfer kinetics, which is critical for the adsorption of macromolecular intermediates and target products [3]. Furthermore, the structural properties of the biomass precursor itself fundamentally determine the development of pore structure; for example, cellulose-based hydrogels typically self-assemble to form a uniform hierarchical micro-mesoporous structure [14]. Importantly, the inherent inorganic constituents present in some biomass feedstocks can act as in situ self-templates, eliminating the need for exogenous additional templates [15]. After the first sol–gel transition, the as-formed hydrogel requires a thermal curing treatment (60–80 °C) to induce particle agglomeration and enhance the cross-linked matrix, and then sufficient solvent displacement to remove the residual aqueous phase prior to drying.

The subsequent drying process is a critical step determining whether the aerogel can retain its intact texture and hierarchical pore structure. Advanced drying technologies such as supercritical drying and freeze-drying are widely employed for the preparation of high-performance carbon aerogels. These methods can eliminate the liquid-vapor phase interface and effectively eradicate capillary stress, thereby preserving the highly connected open-pore skeleton, ensuring smooth ion transport, and maximizing specific surface area [16]. In contrast, atmospheric pressure drying features the advantages of straightforward scale-up and low commercialization cost. However, extremely strong capillary forces are generated during solvent evaporation, which often causes severe structural shrinkage and pore collapse. Such structural damage substantially reduces the available specific surface area and ultimately leads to a significant decline in the electrochemical and adsorption properties of the carbon materials.

In the final stage, the dried organic aerogel is subjected to controlled pyrolysis (300–2500 °C) under an inert atmosphere to remove volatile heteroatom components and carbonize the polymer skeleton into a highly porous conductive carbon matrix. Pyrolysis temperature is the most critical thermodynamic parameter that determines the texture and electrical properties of carbon aerogels. Moderate pyrolysis temperatures (700–1000 °C) facilitate the generation of a large number of micropores and mesopores, significantly increasing the specific surface area and total pore volume [17]. However, when the temperature exceeds the optimal threshold (usually greater than 1000 °C), local collapse of the fine porous skeleton occurs, resulting in a noticeable decrease in pore volume. In addition, ultra-high temperature heat treatment (>2000 °C) induces deep structural ordering and graphitization, and this process greatly improves electrical conductivity by reconstructing the sp^2^-hybridized carbon network, but severely sacrifices the textural properties of the material [17]. In summary, to promote the industrial application of biomass-derived carbon aerogels, a holistic optimization scheme is required: adopting low-pretreatment feedstocks, rationally regulating catalyst ratios to achieve microstructural control at low cost, selecting energy-efficient drying processes, and customizing the heat treatment process according to practical application requirements, so as to achieve an optimal balance between specific surface area and electrical conductivity.

#### 2.1.1. Precursor Gelation

The sol–gel process serves as a critical prerequisite for constructing three-dimensional continuous organic networks. This process enables dispersed macromolecular or small-molecule precursors to form self-supporting wet gels through intermolecular interactions or chemical reactions, providing a topological framework for subsequent drying and pyrolysis. If the crosslinking degree of the gel network is insufficient, or a large number of defective junctions exist, the framework is prone to collapse and shrink during solvent removal and high-temperature treatment, making it difficult to obtain monolithic carbon aerogels [13].

Natural polymers such as cellulose, lignin, and chitosan are currently the primary raw materials for bio-based carbon aerogels [14,18]. Physical crosslinking is achieved through molecular chain entanglement, hydrogen bonding, and ionic coordination. In the case of cellulose nanofibers, abundant surface hydroxyl groups enable them to form gels directly in water. This preparation route features mild conditions and is environmentally friendly. However, the resulting network exhibits poor mechanical properties and is prone to swelling or dissociation under drying stress. Its limited ability to resist the stress generated during drying reduces structural stability. Therefore, in recent years, numerous studies have introduced chemical crosslinking strategies. Reagents such as boric acid, polyvinyl alcohol, and sodium tripolyphosphate are used to construct coordination bonds or covalent crosslinking nodes, thereby strengthening the mechanical stability of the gel network [14].

#### 2.1.2. Drying

During the drying process, the solvent must be removed while the three-dimensional network structure is retained. The gas–liquid interface formed during solvent evaporation generates large capillary stress, which is the primary cause of framework shrinkage and pore structure collapse [19]. Three typical drying techniques are adopted for aerogel preparation, including freeze-drying, supercritical drying, and ambient pressure drying.

##### Freeze-Drying

Freeze-drying consists of two sequential processes, including low-temperature freezing and vacuum sublimation. During the process, the solvent forms ice crystals that occupy the pore space, which then sublimate directly into vapor. This process avoids the formation of a gas–liquid interface and reduces the structural damage caused by capillary action [19]. The freezing conditions, including the cooling rate and freezing direction, can be adjusted according to the requirements of the precursor system, providing flexibility in controlling the drying process [20]. For instance, Bao et al. developed elastic carbon nanofiber aerogels (CBCNT) with a well-defined porous framework using bacterial cellulose (BC) as the raw material via unidirectional freeze-drying technology [21]. However, this process has disadvantages, including long duration, high energy consumption, and potential irreversible deformation of flexible skeletons. In addition, the freezing and sublimation steps require relatively long processing times, which may limit the efficiency of large-scale preparation.

##### Supercritical Drying

**Figure 2:**
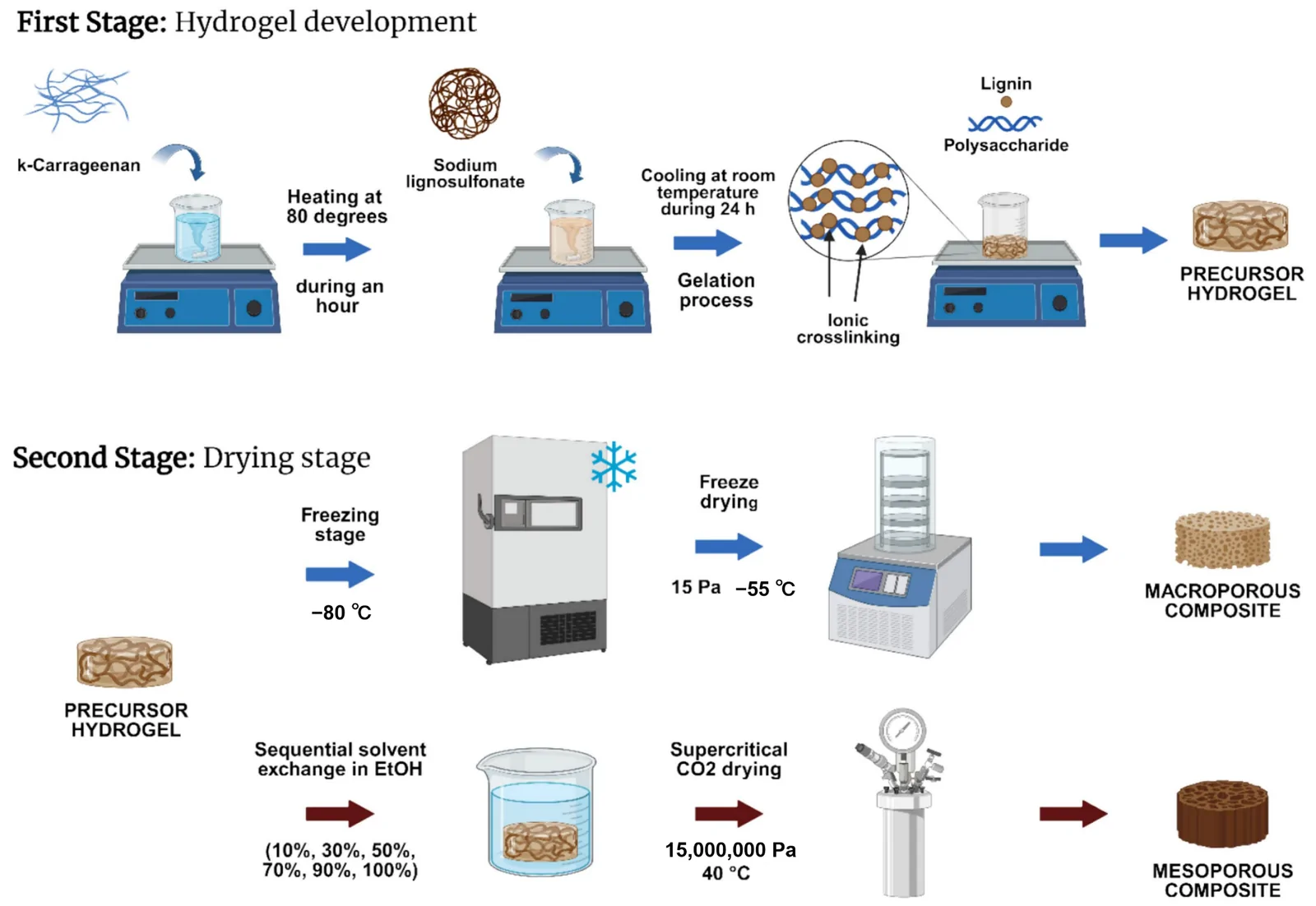
Preparation of lignin-polysaccharide porous materials by different drying methods [22]. Copyright 2025, Elsevier.

Supercritical drying is an effective approach for minimizing structural shrinkage during the drying process [16]. Solvent replacement is sequentially completed by first displacing water in the wet gel with ethanol, and then further displacing ethanol with liquid CO_2_. Under supercritical conditions, CO_2_ transforms into a supercritical fluid, where the gas–liquid interface nearly disappears, and interfacial tension approaches zero. Consequently, the gel skeleton is barely subjected to capillary stress, yielding products with low shrinkage. However, this method requires high-pressure equipment with high capital costs, the solvent exchange process is time-consuming, and it is challenging to achieve large-scale industrial scaling up. Alvarez-Valcarce systematically compared supercritical CO_2_ drying and freeze-drying processes (Figure 2) using sodium lignosulfonate and κ-carrageenan as precursors, with the comparison focusing on the morphology and pore structure of the prepared materials. The comparison demonstrates the feasibility of supercritical CO_2_ drying for preparing biomass-derived porous materials [22].

##### Ambient Pressure Drying

Ambient pressure drying removes the solvent by evaporation at atmospheric pressure. This method features simple equipment and low costs, representing a process route with the most promising prospects for industrial production [23]. The primary challenge of this method lies in the continuous formation of gas–liquid interfaces, which generates large capillary stress that may cause the gel to shrink or crack. Solvent replacement with low-surface-tension solvents and surface hydrophobic modification can effectively reduce capillary forces. Wei et al. fabricated TOCNF/MS (TMS) wet gels using TEMPO-oxidized cellulose nanofibers (TOCNF) and MXene sediment (MS) as raw materials via a dual crosslinking strategy combining hydrogen bonding and zirconium ion (Zr^4+^) crosslinking [23]. Ethanol was introduced prior to ambient pressure drying to reduce the surface tension of the solvent, further weakening the capillary force during the drying process. Zhang et al. fabricated flexible cellulose aerogels (A-CNFA) using a refrigerator-assisted freezing process combined with ambient pressure drying [24]. They adopted an isopropyl alcohol (IPA) solvent replacement protocol, which effectively mitigated the structural collapse and volume shrinkage that commonly occur during ambient pressure drying.

However, quantitative comparison of energy consumption, processing time, and scalability is more difficult because these parameters strongly depend on sample size, solvent system, equipment configuration, and processing scale. In general, freeze-drying requires prolonged freezing and vacuum sublimation (approximately 24–48 h) [25], whereas supercritical drying involves solvent exchange and high-pressure operation, resulting in greater process complexity and energy consumption. Ambient-pressure drying requires simpler equipment and has greater potential for large-scale production, but generally results in greater shrinkage and lower surface-area retention. Therefore, drying methods should be evaluated by considering structural shrinkage, energy consumption, processing time, and scalability, while recognizing that these parameters are strongly dependent on processing conditions.

##### Spray Drying

Spray drying is another rapid drying technique that converts precursor suspensions into dry particles through atomization and hot-gas-assisted solvent evaporation. Compared with freeze-drying and supercritical drying, it offers shorter processing times, simple operation, and better scalability, making it attractive for large-scale preparation of biomass-derived porous carbon precursors. However, rapid solvent removal and thermal exposure may promote particle aggregation and structural densification, which can compromise the preservation of the three-dimensional porous network [26]. Therefore, spray drying is particularly suitable for the preparation of particulate porous carbon materials, while additional structural design may be required when highly open three-dimensional monolithic structures are desired.

#### 2.1.3. Carbonization and Heat Treatment

**Figure 3:**
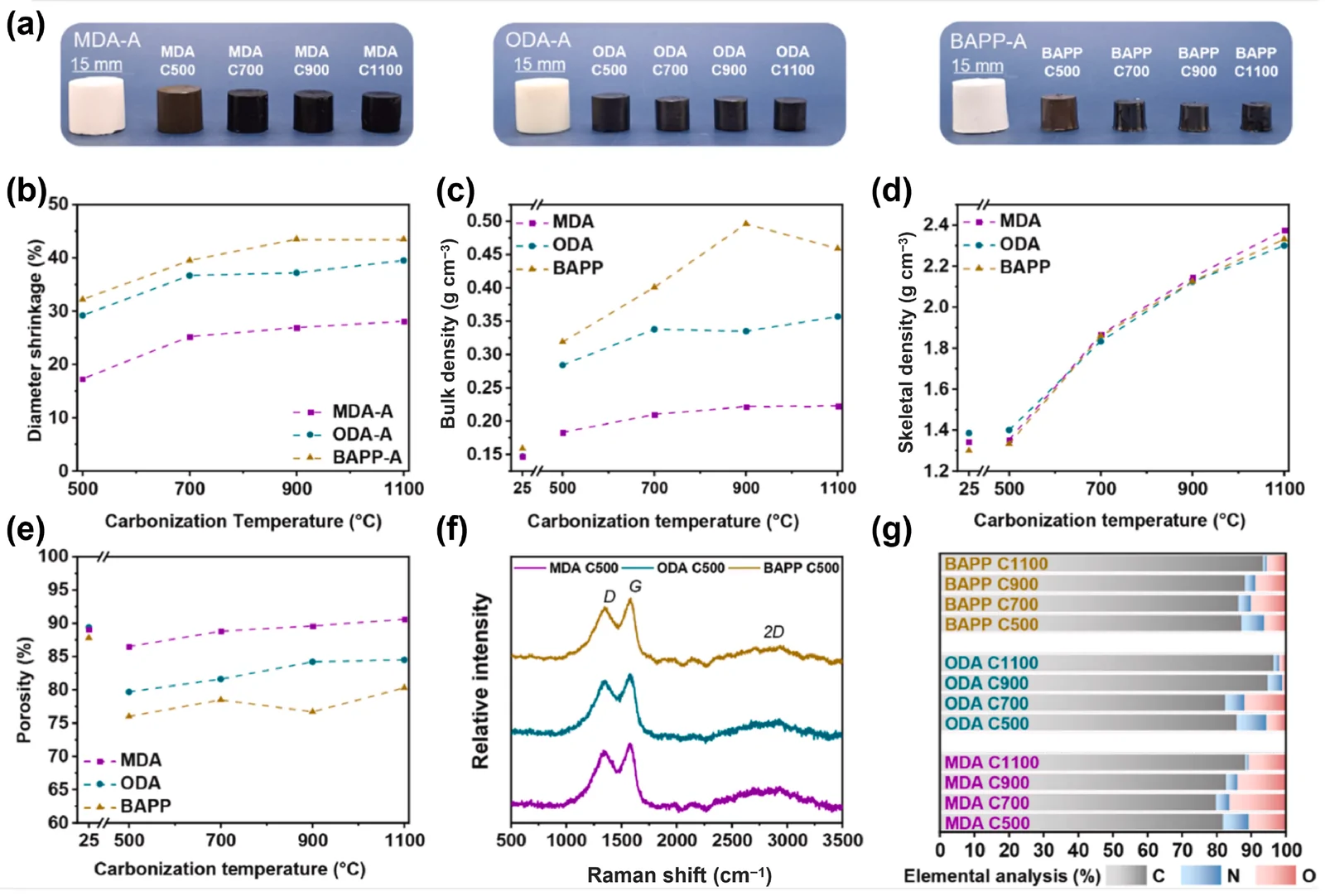
(**a**) Photographic images of MDA-A, ODA-A, BAPP-A and their corresponding carbonized analogs (scale bar 15 mm). (**b**) Diameter shrinkage. (**c**) Bulk density. (**d**) Skeletal density. (**e**) The porosity of carbon aerogels. (**f**) Raman spectra for the samples carbonized at 500 °C. (**g**) Elemental analysis from XPS [27]. Copyright 2026, Elsevier.

Carbonization converts organic aerogels into carbon aerogels and must be conducted in an inert atmosphere such as nitrogen or argon [16]. A heating rate of 1–5 °C min^−1^ and a holding time of 1–3 h are generally adopted to ensure sufficient progress of the pyrolysis reaction. Pyrolysis proceeds stepwise at different temperatures. Below 300 °C, the process is dominated by dehydration and small molecule desorption. When the temperature ranges from 300 °C to 600 °C, polymer chains decompose and release a large quantity of volatiles, with mass loss reaching 50–70%. When the temperature exceeds 600 °C, aromatization occurs in the carbon skeleton, and a graphite-like structure is gradually formed. Carbonization at 400–600 °C retains the porous, low-density structure but features a low graphitization degree and poor electrical conductivity. Carbonization at 800–1000 °C achieves a balance between pore structure and electrical conductivity, making the resulting material suitable for the fields of energy storage and flexible sensing. Pantazidis et al. prepared heteroatom-doped carbon aerogels using aromatic poly(imine-cyanurate) networks as precursors via a sol–gel process combined with pyrolysis [27]. Obvious dimensional shrinkage was observed in the samples after carbonization (Figure 3a). Although all samples exhibited similar trends in mass loss, the degree of shrinkage varied with the molecular structure of the precursor (Figure 3b). The resulting carbon aerogels exhibited bulk densities of 183–496 mg cm^−3^ (Figure 3c), while their skeletal densities remained almost consistent (Figure 3d). The porosity of the products fell in the range of 76% to 90% (Figure 3e), showing an inverse correlation with bulk density. Raman spectra revealed that the G-band (~1580 cm^−1^) and D-band (~1350 cm^−1^) existed in all samples, confirming the coexistence of ordered graphitic domains and disordered carbon regions (Figure 3f). The I_D_/I_G_ ratio varied with pyrolysis temperature, indicating a competitive relationship between defect formation and high-temperature graphitization (Figure 3g). When the temperature exceeds 1200 °C, the graphitization degree and electrical conductivity increase significantly, but micropores collapse, leading to a decrease in specific surface area [17]. Yan et al. investigated electrospun PAN-derived carbon nanofiber aerogels and comparatively analyzed the effects of different heat-treatment temperatures on their structure and mechanical properties [28]. Carbon nanofiber aerogel (CNFA-1200) treated at 1200 °C exhibited excellent superelasticity, whereas local structural collapse occurs in the material when heat-treated at 1400 °C, and its stress under 50% compressive strain is lower than 2 kPa.

In addition to conventional pyrolysis, hydrothermal carbonization (HTC) provides an alternative route for converting biomass into carbon-rich materials under hydrothermal conditions. Unlike conventional dry pyrolysis, HTC can directly process wet biomass in water, with hydrolysis, dehydration, decarboxylation, and aromatization occurring during the hydrothermal reaction. The resulting hydrochar generally retains abundant oxygen-containing functional groups, while its carbon structure can be further modified by subsequent heat treatment or activation [29]. This approach is particularly attractive for biomass-derived carbon materials because it reduces dependence on energy-intensive drying and provides an alternative route for regulating carbon structure and surface chemistry. Microwave-assisted pyrolysis provides another alternative heat-treatment strategy by using microwave irradiation to generate rapid volumetric heating. Compared with conventional external heating, this method can shorten the heating process and facilitate the rapid conversion of biomass precursors into carbon materials. Zhou et al. prepared biomass-derived carbon aerogels from α-cellulose and sodium lignosulfonate through microwave pyrolysis [30]. The introduction of sodium lignosulfonate promoted the formation of carbon vacancies and sulfur-containing structures in the carbon aerogel, demonstrating that microwave pyrolysis can not only accelerate carbonization but also regulate the defect and surface chemistry of biomass-derived carbon aerogels.

### 2.2. Regulation of Pore Structure

Regulating the pore structure of biomass-derived carbon aerogels is a fundamental prerequisite that determines the apparent catalytic activity and mass transfer kinetics of electrocatalytic materials [2]. Electrocatalytic reactions, such as the oxygen reduction reaction (ORR), involve complex multi-step electron transfer and mass transport processes at the gas–liquid-solid three-phase interface. Among these, micropores (<2 nm) can provide an extremely high specific surface area, which is used for anchoring and exposing active sites at high density (such as M-N-C catalytic centers, heteroatom-doped sites, and intrinsic topological defects) [5]. However, a single microporous network easily leads to low accessibility of active sites and a sharp increase in local mass transfer resistance. The introduction of mesopores (2–50 nm) not only constructs diffusion channels with low resistance to accelerate the rapid migration of dissolved oxygen (O_2_) to active sites and the timely discharge of products (H_2_O or OH^−^), but also significantly improves access to internal active sites. In addition, macropores (>50 nm) act as reservoirs for electrolytes and buffers for mass transfer, ensuring thorough mass transfer at high current densities. Therefore, the elaborate design and construction of a highly interconnected micropore-mesopore-macropore hierarchical porous architecture is of enormous scientific significance for breaking mass transfer bottlenecks, maximizing site utilization efficiency, and improving the kinetics of the oxygen reduction reaction [2]. Importantly, the design of such hierarchical porous architectures does not imply the existence of a universal optimal pore-size distribution or relative pore-volume ratio for zinc–air battery electrodes. Micropores provide high surface area and abundant sites for catalytic active centers, whereas mesopores and macropores facilitate oxygen transport, electrolyte infiltration, and product removal during ORR/OER. However, excessive microporosity may limit active-site accessibility and increase mass-transfer resistance. Therefore, the appropriate balance among micro-, meso-, and macropores should be tailored to the catalyst loading, electrode thickness, electrolyte environment, and operating current density, rather than determined solely by maximizing the specific surface area.

To achieve precise regulation and hierarchical optimization of the pore structure of carbon aerogels, researchers usually adopt a variety of combined strategies such as physical/chemical activation, template method, and in situ foaming. Chemical activation methods (e.g., impregnation with KOH, ZnCl_2_, or H_3_PO_4_) can construct highly developed micropores and abundant mesopores in the carbon skeleton through selective etching at high temperatures and the release of gases (such as CO_2_ and H_2_O), and significantly broaden the pore size distribution; physical activation (e.g., annealing in CO_2_ or water vapor atmosphere) can selectively open pores while maintaining the strength of the skeleton [31]. The template regulation method includes using colloidal SiO_2_ as hard templates or block copolymers (such as P123 and F127) as soft templates, thereby producing a mesoporous/macroporous network with highly uniform and predictable pore sizes. In addition, combining the intrinsic chemical properties of biomass precursors, adopting in situ self-templating and in situ foaming strategies (for example, introducing nitrogen-rich blowing agents such as urea and melamine, or utilizing the microscopic templating effect of endogenous inorganic salts in biomass) can enable the in situ opening of closed pores through the escape of volatile components during pyrolysis, eliminating the need for tedious post-treatment template removal steps [15]. Through the synergistic integration of sol–gel kinetics control, directional drying protection, and annealing/activation processes, customized regulation of the microscopic pore size, pore volume, and three-dimensional connectivity of carbon aerogels can be achieved, thereby meeting the microscopic structure requirements of high-performance ORR electrocatalysts.

#### 2.2.1. Regulation of Pores by Drying Methods

Drying conditions strongly influence the final pore architecture of carbon aerogels, including pore-size distribution, pore connectivity, pore orientation, and framework density [32]. Therefore, the effects of different drying methods are mainly discussed in terms of specific surface area, pore structure, and pore-size distribution. Supercritical drying generally preserves a highly open and interconnected meso-macroporous framework, whereas ambient pressure drying tends to produce a denser structure with greater shrinkage and a lower proportion of accessible mesopores [16]. These differences in pore architecture can influence the accessibility of internal active sites and the transport of oxygen, electrolyte, and reaction products, and are therefore relevant to the performance of porous air electrodes [33]. Control experiments were carried out using the same cellulose aerogel fiber precursor. The results show that the shrinkage rates corresponding to ambient pressure drying, freeze drying and supercritical drying are approximately 48.7%, 36.3% and 2.6%, respectively, and the corresponding specific surface areas are 4.78, 175.21 and 371.94 m^2^ g^−1^ [34]. These results indicate that, under comparable conditions, supercritical drying provides better structural retention and BET surface-area preservation.

Carbon aerogels prepared by ambient pressure drying are dominated by microporous structures, and their mesopore area and specific surface area are significantly lower than those of samples obtained by supercritical or freeze-drying [23]. The resulting framework generally exhibits greater shrinkage and lower pore retention, leading to a denser and partially collapsed structure. To address this issue, researchers have developed strategies such as solvent replacement with low-surface-tension solvents (e.g., replacing water with ethanol or acetone) and gel skeleton reinforcement. These approaches can reduce structural shrinkage and improve framework integrity [24]. In addition, Men et al. prepared environmentally friendly nanoporous bulk carbon aerogels via ambient pressure drying using chitosan as both the crosslinking agent and supporting template [35]. The obtained carbon aerogels exhibited a pore-size range of 43–107 nm, indicating the formation of a relatively fine nanoporous structure. The relatively dense framework and limited pore retention after ambient pressure drying are consistent with the lower specific surface area generally observed for this drying method. However, even after solvent exchange, ambient pressure drying still struggles to completely avoid structural shrinkage, and the retention of the pore structure remains relatively low. In contrast, supercritical drying can effectively preserve the porous framework and reduce structural shrinkage. Men et al. incorporated self-crosslinked chitosan into the resorcinol-formaldehyde system to improve the mechanical strength of the gel network, and subsequently prepared carbon aerogels via supercritical CO_2_ drying [33]. The resulting carbon aerogels exhibited a pore-size range of 9–130 nm, with shrinkage during drying as low as 1.51%.

Freeze-drying provides additional control over pore structure through ice-crystal formation and growth. Hu et al. investigated the effects of conventional freeze-drying and step-freeze-drying on the pore structure of carbon aerogels [36]. For conventional freeze-drying, the carbon aerogel exhibited a specific surface area of 505.05 m^2^ g^−1^ and a predominantly mesoporous structure. Step-freeze-drying further increased the specific surface area to 916.55 m^2^ g^−1^ and produced a finer pore structure, indicating that the freezing process can regulate ice-crystal growth and consequently the pore-size distribution of carbon aerogels. In contrast, directional freeze-drying provides an additional degree of control over pore orientation and anisotropy. By controlling ice-crystal growth, highly oriented macroporous channels can be generated along the freezing direction [37]. These aligned channels provide low-tortuosity pathways for electrolyte or gas transport, while the precursor framework and subsequent carbonization determine the accompanying meso- and microporous structures. Consequently, directional freeze-drying can generate hierarchical and anisotropic architectures in which pore orientation becomes an additional structural parameter beyond pore size and specific surface area.

**Figure 4:**
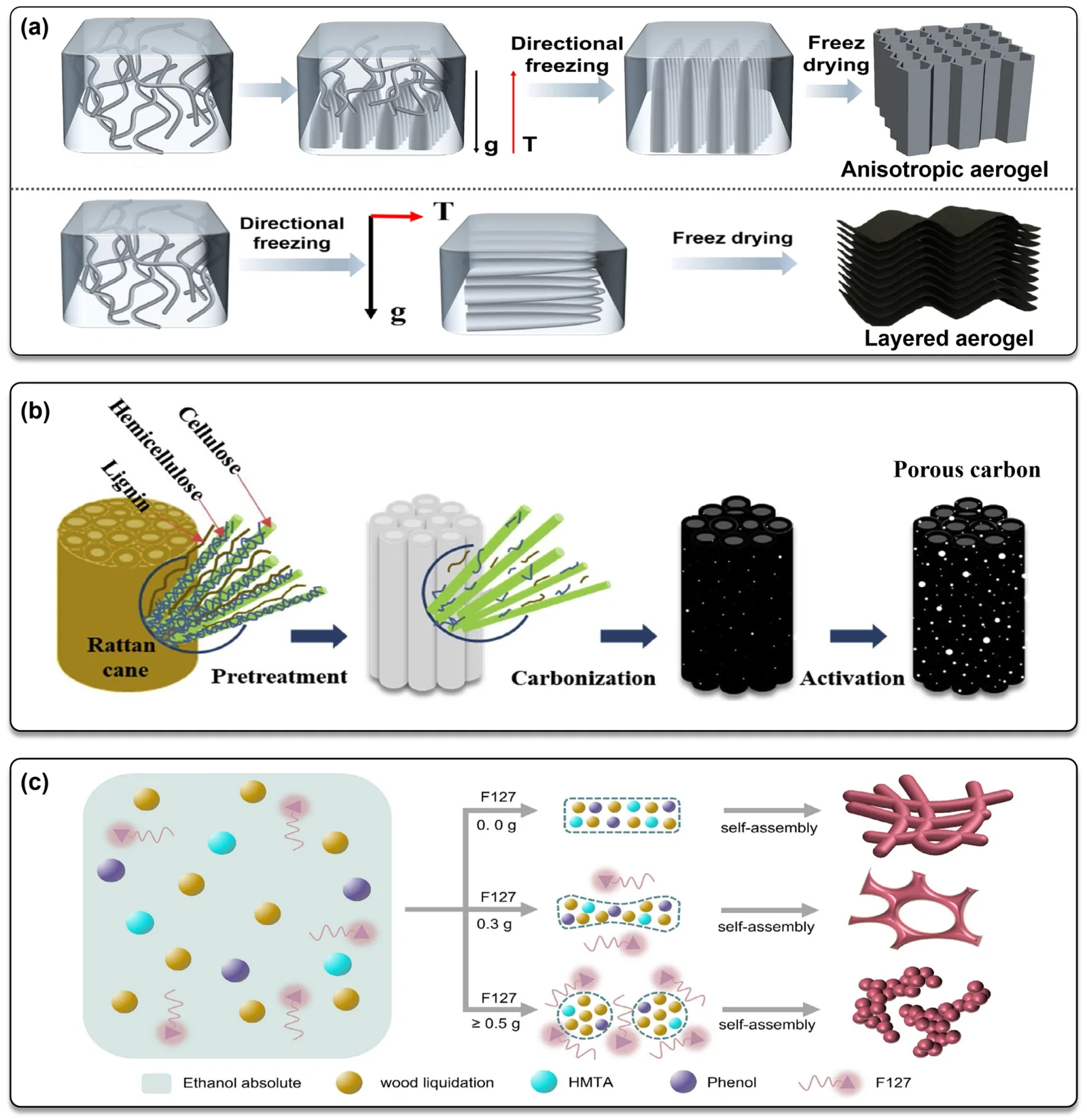
(**a**) Formation of honeycomb pore structure and formation of layered pore structure [38]. Copyright 2025, Elsevier. (**b**) Schematic illustration of the synthesis of a-RCA [42]. Copyright 2020, Elsevier. (**c**) Schematic diagram of using F127 as a soft template to prepare size-controllable biomass-based aerogel [43]. Copyright 2025, Elsevier.

Directional freeze-drying can generate distinct pore architectures depending on ice crystal growth behavior, with honeycomb and layered structures being the most representative (Figure 4a). Honeycomb architectures, derived from columnar or cellular ice crystals, are characterized by regular and interconnected cellular pores [38]. When external stress is applied perpendicular to the pore direction, this structure can distribute the stress efficiently and suppress local collapse, which leads to excellent mechanical robustness and cycling stability, rendering them highly suitable for pressure sensing. However, highly uniform pore size may limit the deformation gradient and narrow the detectable pressure range. In contrast, the layered pore structures originate from the directional growth of lamellar ice crystals, thereby forming an architecture of stacked and undulating lamellar structures. Under compression, the varied interlayer spacings enable differential deformation, which can lead to distinct resistance changes across a wide pressure range and thus improve the sensing sensitivity [39]. While these lamellar structures are more prone to interlayer collapse when subjected to high compressive loads, they usually require adjustments to the sol composition or the synergistic effects of multicomponent systems for reinforcement. Overall, directional freeze-drying can precisely control ice-crystal growth and pore orientation, offering an effective approach to constructing honeycomb-like and multi-layer architectures. This strategy is essential for achieving the synergy between structure and performance in carbon aerogels for advanced electrochemical devices and pressure sensors [40].

Overall, the three drying methods exhibit distinct effects on pore structure. Ambient pressure drying generally results in greater shrinkage, lower specific surface area, and poorer retention of accessible pores. Supercritical drying provides better preservation of the original porous framework, resulting in higher specific surface area, smaller characteristic pore sizes, and better pore connectivity [25]. Freeze-drying provides relatively high specific surface area and enables the formation of mesoporous or hierarchical pore structures, while pore-size distribution can be further regulated by controlling ice-crystal growth. For zinc–air batteries, a high specific surface area and accessible micropore/mesopore network can increase active-site accessibility, whereas interconnected meso- and macropores facilitate oxygen and electrolyte transport [41]. For piezoresistive sensors, pore size, pore connectivity, and pore orientation influence framework deformation and the evolution of conductive contacts, thereby affecting sensitivity, working range, and mechanical stability [39].

#### 2.2.2. Activation and Templating Methods

The chemical activation process for regulating pore structure typically entails mixing biomass with an activator in a certain proportion, followed by an impregnation process and the subsequent conducting of pyrolysis treatment in an inert atmosphere [44]. During this thermal treatment process, the activator promotes the formation of pores in the carbon skeleton. The effectiveness of activation depends on various factors, such as the type of activator, dosage, and temperature. Commonly used activators include ZnCl_2_, K_2_CO_3_, KHCO_3_, H_3_PO_4_, and KOH [45]. This method facilitates the rapid generation of carbon materials with abundant pores and is particularly suitable for preparing bio-based carbon aerogels with high specific surface areas and tunable pore-size distributions. For cellulose-derived carbon aerogels, Yang et al. successfully constructed a micro-, meso-, and macroporous hierarchical network structure through intensive etching with potassium hydroxide, and dramatically increased the specific surface area of the activated sample CA-KOH-3 from 616.97 m^2^ g^−1^ to 1709.62 m^2^ g^−1^ [46]. The formation of pores across multiple length scales indicates that KOH activation can simultaneously increase accessible surface area and broaden the pore-size distribution. Cui et al. successfully prepared bamboo-fiber-derived carbon aerogels via high-temperature activation with potassium hydroxide, and the specific surface area of the obtained carbon aerogel reached as high as 2503.80 m^2^ g^−1^ [47]. The KOH activation promoted the formation of abundant mesoporous structures on the pore walls, demonstrating that the activation conditions can regulate not only the specific surface area but also the micro/mesoporous characteristics of the carbon aerogel. Yang et al. obtained the initial rattan carbon aerogel (RCA) by conducting chemical pretreatment, freeze-drying, and carbonization of the rattan strips [42]. Subsequent KOH activation was performed on the as-synthesized RCA, which generated a large number of micropores and mesopores, significantly expanded the pore structure, and increased the specific surface area (Figure 4b). The specific surface area of the non-activated RCA was approximately 947 m^2^ g^−1^, while that of the three-dimensional nanosheet carbon aerogel (a-RCA) formed after activation had a significantly increased specific surface area of 2284 m^2^ g^−1^, with a total pore volume of 4.27 cm^3^ g^−1^. Such a high specific surface area and hierarchical pore structure significantly enhanced the oxygen diffusion and electrolyte permeation capabilities, thereby enhancing the catalytic activity and cycle stability of the air cathode in zinc–air batteries. Therefore, the pore structure generated by KOH activation should maintain an appropriate balance between micropores, which contribute to accessible surface area, and mesopores, which facilitate oxygen and electrolyte transport. However, excessive microporosity may reduce the accessibility of internal active sites and increase mass-transfer resistance, indicating that a higher specific surface area does not necessarily lead to improved catalytic performance [41].

Black liquor, a byproduct of pulp production, is considered an ideal precursor for porous carbon due to its high content of inorganic salts (30–35 wt%, mainly Na and K compounds), which can act as an in situ self-activating agent during carbonization. This intrinsic self-activation proceeds via a distinctive reaction pathway. At elevated temperatures, native alkali metal salts contained in black liquor are reduced into metallic Na and K species. These active metal substances further engage in redox reactions with the carbon skeleton, etching the carbon network to generate abundant micro- and mesopores [15,48]. Unlike conventional chemical activation that relies on external activators such as KOH or ZnCl_2_, this inherent activation avoids the use of additional chemical reagents and streamlines the overall fabrication procedure. This intrinsic self-activation enables direct valorization of raw black liquor into porous carbons with surface areas of 700–1000 m^2^ g^−1^ [49]. Meanwhile, Kraft lignin isolated from black liquor has been converted into carbon aerogels with an ultrahigh specific surface area of 3742 m^2^ g^−1^ [50], demonstrating the significant potential of lignin-derived precursors. In these materials, the development of micro- and mesopores provides both accessible surface area and transport pathways. Thus, self-activation of biomass-derived precursors can generate hierarchical pore structures without the direct addition of conventional chemical activators, which is beneficial for applications requiring both surface accessibility and mass transport.

The physical activation process for regulating pore structure is a two-stage procedure. Initially, the raw material is pre-carbonized at a medium temperature (400–800 °C). Subsequently, at a higher temperature (800–1200 °C), CO, CO_2_, steam, or air is introduced to react with the carbonaceous framework. This reaction effectively etches the carbon to form pores [51]. By adjusting the activation temperature and gas type, this approach enables tailored synthesis of materials with varying pore size distributions and specific surface areas. Compared with chemical activation, physical activation can avoid the use of corrosive reagents, making it a more environmentally friendly process. Zu et al. prepared pristine carbon aerogels (CA-SCD) using nanocellulose as the precursor via supercritical drying and carbonization [31]. Subsequently, ACA-SCD was fabricated through a CO_2_ physical activation process, in which a gas–solid reaction between CO_2_ and the carbon skeleton occurs at high temperature. During the activation process, CO_2_ gently etches the carbon walls, thereby introducing a large number of micropores and forming a certain proportion of mesopores. After the activation treatment of CO_2_, the specific surface area of the carbon aerogel significantly increased from 892 m^2^ g^−1^ to 1873 m^2^ g^−1^, while the total pore volume increased from 1.80 cm^3^ g^−1^ to 2.65 cm^3^ g^−1^. Its pore structure transformed from a relatively dense carbon framework to a hierarchical pore network dominated by micropores with a synergy between micropores and mesopores. This result indicates that the physical activation of CO_2_ can effectively replace chemical activators and is an important method for constructing carbon aerogels with high porosity. Thus, CO_2_ activation mainly promotes the development of micropores while retaining mesoporous transport pathways, resulting in simultaneous increases in specific surface area and pore volume.

The templating approaches for regulating pore structure can be divided into hard-templating [52] and soft-templating [53]. Both of these methods enable the preparation of carbon materials with ordered pores, such as mesopores. The hard-templating method involves using materials with specific nanostructures as templates (e.g., SiO_2_ nanospheres, zeolites). The biomass carbon source is injected into the pore channels of the template, followed by carbonization treatment. The template is subsequently removed via etching with HF or NaOH, yielding a carbon material that is a reverse replication of the template structure. In contrast, the soft-templating method uses amphiphilic block copolymers as soft templates. After these templates self-assemble with the carbon precursor, an ordered mesoporous structure is formed during the carbonization process. Compared with activation, templating provides more direct control over pore size and pore-size distribution, particularly for ordered mesoporous structures. Sun et al. prepared lignocellulose-based carbon aerogels (WCA-F_x_) using pluronic F127 as a soft template [43]. Driven by the dominant effect of the soft template, the specific surface area of WCA-F_x_ reached as high as 1209.65 m^2^ g^−1^, of which 83.67% originated from micropores. Its structure and morphology can also be controlled by adjusting the content of F127 (Figure 4c). This result shows that F127 can regulate the development and distribution of pores while maintaining a high accessible surface area, providing a route for tailoring the pore architecture according to the requirements of mass transport and surface accessibility.

Overall, activation and templating methods can construct carbon aerogels with highly developed, hierarchical, and tunable porous architectures, characterized by increased specific surface area, regulated pore-size distribution, and improved pore connectivity. These structural features enhance surface accessibility and mass transport, thereby facilitating active-site utilization and reactant transport in zinc–air batteries, while providing an appropriate deformation space and conductive-network evolution for piezoresistive sensing. Thus, regulating the pore architecture through activation and templating can effectively tailor the structural and functional properties of carbon aerogels for different applications.

## 3. Doping Engineering of Bio-Based Carbon Aerogels for Zinc–Air Batteries

Bio-based carbon aerogels often require further structural and compositional modification to achieve competitive oxygen electrocatalytic performance [2]. This section summarizes two major active-site engineering strategies, including heteroatom doping and non-noble metal incorporation. The effects of different doping approaches, coordination environments, and structural regulation on the catalytic performance of biomass-derived carbon aerogels are discussed. The catalytic performance of bio-based carbon aerogels is closely associated with both the intrinsic characteristics of active sites and their accessibility within the porous framework. Hierarchical pore structures play important roles in regulating electrocatalytic processes. Micropores can increase accessible surface area and facilitate the exposure of defect-associated or atomically dispersed active sites, while mesopores and macropores are beneficial for electrolyte penetration, oxygen diffusion, and mass transport [54]. Therefore, rational regulation of pore architecture and active-site construction should be considered as complementary strategies for optimizing oxygen electrocatalytic performance.

### 3.1. Heteroatom Doping Engineering

**Figure 5:**
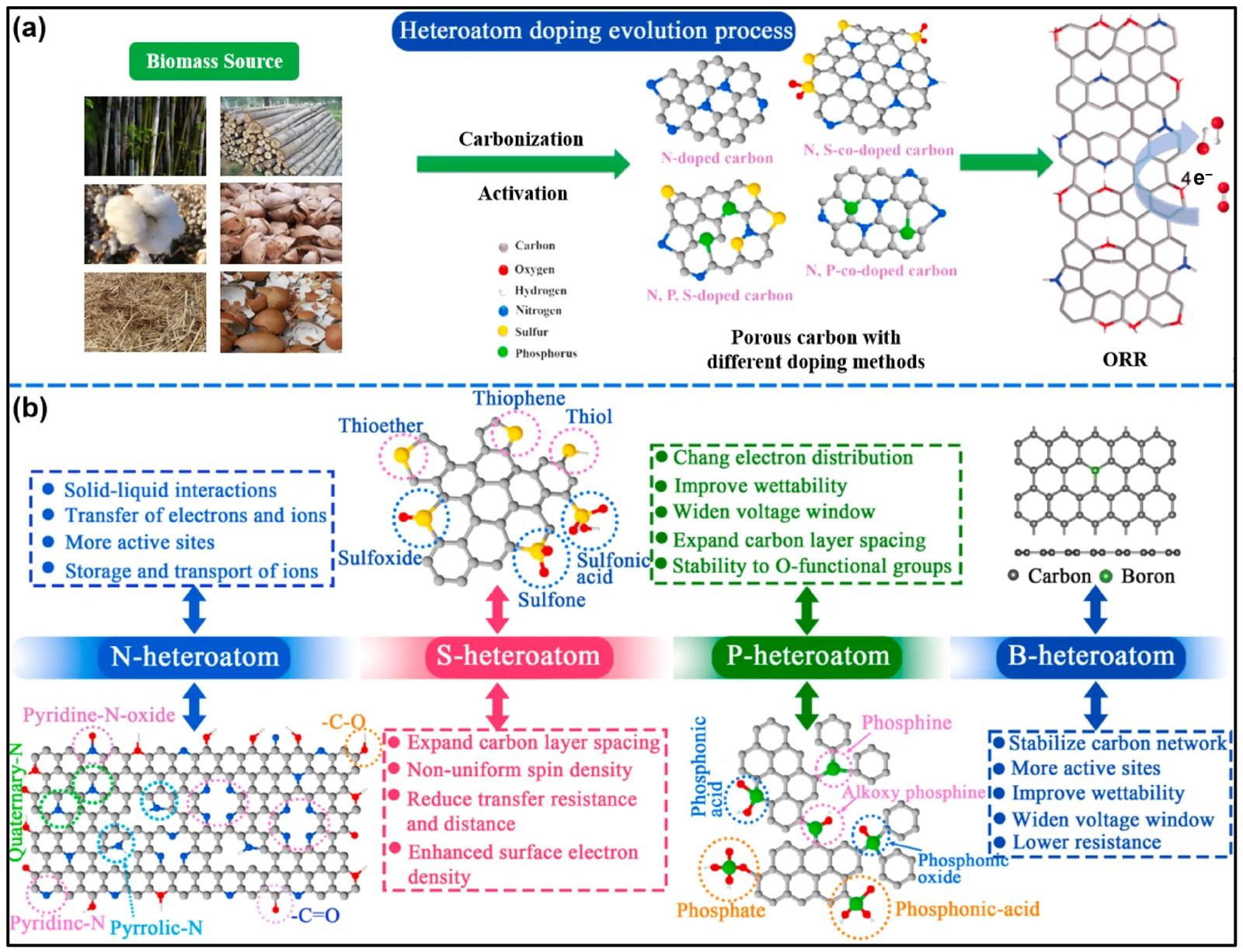
(**a**) Molecular structure changes during the conversion of biomass resources to heteroatom-doped carbon materials. (**b**) Effects of heteroatom doping on the structure of biomass-based carbon materials [55]. Copyright 2024, Elsevier.

Doping engineering is essential for overcoming the intrinsically low catalytic activity of pristine biomass-based carbon aerogels and for constructing highly active sites for oxygen electrocatalysis. Biomass resources, such as bamboo, wood, cotton, nutshells, straw, and eggshells, can be converted into heteroatom-doped porous carbon through carbonization/activation under an inert atmosphere, ultimately enabling the 4e^−^ ORR pathway in alkaline media (Figure 5a). Fan et al. summarized the main doping strategies and functional mechanisms of N, S, P, and B heteroatoms in carbon materials (Figure 5b) [55]. Among these, nitrogen doping is the most extensively studied. It serves as the core strategy for regulating the electronic structure and active site distribution of carbon materials. Different nitrogen species, including pyridinic N, pyrrolic N, graphitic N, and oxidized N, play critical roles in catalytic activity [56]. In situ and post-treatment doping are two common approaches for introducing nitrogen into carbon frameworks. In situ doping during carbonization generally enables more uniform incorporation of dopants and facilitates the formation of stable active sites. In contrast, post-treatment doping offers greater flexibility in adjusting surface chemical composition, although the dopant distribution may be less uniform due to limited diffusion within the carbon matrix [57]. Co-doping with sulfur, phosphorus, or boron can further regulate the electronic structure and surface chemistry of carbon frameworks. Generally, sulfur and phosphorus co-doping mainly modifies the local electronic environment of carbon atoms and catalytic sites, while boron doping tends to introduce electron-deficient characteristics due to its lower electronegativity compared with carbon. The selection of different heteroatom combinations depends on their influence on active-site formation, charge distribution, and intermediate adsorption during oxygen electrocatalysis [58]. These factors synergistically optimize the bifunctional catalytic performance and guide the rational design of bio-based carbon aerogels as high-performance air cathodes for zinc–air batteries.

#### 3.1.1. The Role of Nitrogen Doping

Pristine bio-based carbon aerogels typically exhibit relatively low catalytic activity. Therefore, it is necessary to introduce heteroatom doping, especially nitrogen doping to construct highly active sites [54]. Nitrogen doping is a core strategy for regulating the electronic structure and active site distribution of carbon materials, and it has demonstrated significant advantages in applications such as electrocatalysis, energy storage, and sensing [59,60]. Owing to the higher electronegativity of nitrogen atoms compared to carbon atoms, and the introduction of lone pairs of electrons in their five-valence-electron configuration, N-doping markedly enhances the polarity of the carbon framework and simultaneously improves the electron transport efficiency [54]. Additionally, the incorporation of nitrogen can induce structural defects in the carbon network, modulate the electronic band structure, optimize surface wettability, and accelerate the kinetics of interfacial reactions. To rigorously decouple and independently evaluate the multifactorial origins of the enhanced oxygen reduction reaction/oxygen evolution reaction (ORR/OER) performance induced by heteroatom and metal doping, a targeted experimental characterization framework addressing five core components is required. Specifically, high-resolution XPS and XAS (employing XANES/EXAFS to probe the local coordination environment and oxidation states) are utilized to investigate electronic structure modulation, thereby separating intrinsic charge redistribution from morphological changes. Raman spectroscopy (using intensity ratios to characterize topological/edge defect density), electron paramagnetic resonance (EPR) (for localized carbon vacancy signals), and aberration-corrected high-angle annular dark-field scanning transmission electron microscopy (AC-HAADF-STEM) are employed to quantitatively distinguish defect generation from chemically active sites. Contact angle measurements in the working electrolyte are conducted to characterize wettability enhancement. By comparing the equivalent series resistance and charge transfer resistance obtained from bulk four-point probe measurements or electrochemical impedance spectroscopy (EIS), the electrical conductivity of the solid framework is decoupled from interfacial charge transfer. Finally, the electrochemically active surface area (ECSA) is extracted from double-layer capacitance measurements to decouple the physical specific surface area (obtained from adsorption measurements) from the actually accessible solid–liquid electrochemical interface, thereby avoiding oversimplified attributions regarding the mechanisms of performance enhancement [61].

The incorporation of nitrogen atoms into carbon materials typically yields four distinct bonding configurations, namely pyrrolic nitrogen (N-5), pyridinic nitrogen (N-6), graphitic nitrogen (N-Q), and oxidized nitrogen (Figure 5). Each of these nitrogen types plays a critical role in modulating electrocatalytic reactions, tuning electronic structures, and enhancing catalytic performance. Although extensive studies have confirmed the respective catalytic roles of pyridinic nitrogen, pyrrolic nitrogen, and graphitic nitrogen, it must be prudently recognized that existing ex situ experimental evidence has nearly reached its limit in identifying the true nitrogen-containing active configurations for the oxygen reduction reaction/oxygen evolution reaction (ORR/OER). The principal bottleneck in this field arises from two unavoidable objective circumstances: the coexistence of multiple components and their dynamic transformations. First, conventional thermal doping or pyrolysis strategies inevitably generate heterogeneous mixtures of multiple nitrogen species, making it difficult to isolate and quantitatively characterize the independent contribution of a single configuration. Second, such nitrogen active sites are not chemically steady-state structures. Under the harsh reductive/proton-coupled environment of the oxygen reduction reaction, or the high oxidative potential conditions of the oxygen evolution reaction, nitrogen configurations undergo dynamic structural evolution, including protonation, reversible oxidation (generating oxidized nitrogen species), and even structural degradation and leaching. Consequently, conventional ex situ characterization techniques (such as routine X-ray photoelectron spectroscopy before and after reaction) are incapable of tracking transient active phases at the charged solid–liquid interface. To overcome this methodological bottleneck and definitively identify the true active centers, future research needs to shift toward the preparation of model catalysts with precisely controllable chemical structures (e.g., employing bottom-up molecular precursor design strategies), combined with advanced in situ and operando spectroscopic techniques (such as in situ Raman spectroscopy, Fourier transform infrared spectroscopy, and operando X-ray absorption spectroscopy/X-ray photoelectron spectroscopy), to monitor the real-time dynamic evolution of nitrogen configurations during electrochemical processes [61,62]. In addition, recent studies have reported different correlations between specific N configurations and ORR activity, suggesting that no single N configuration has been universally established as the dominant active site. Therefore, the relationship between the relative abundance of a specific N species and the overall catalytic activity should be considered with caution, as the observed performance may result from the synergistic contributions of multiple N configurations and their surrounding carbon structures. Specifically, pyrrolic nitrogen (N-5) [63] exhibits weak basicity and functions as a Brønsted acid site. It improves the adsorption of intermediates involved in the oxygen reduction (ORR) and oxygen evolution reactions (OER) via hydrogen bonding or dipole-quadrupole interactions, thus boosting catalytic activity. Pyridinic nitrogen (N-6), acting as a Lewis base site, facilitates the adsorption and activation of oxygen molecules through acid-base interactions, thereby accelerating the ORR rate [4]. Graphitic nitrogen (N-Q) embedded in the carbon framework has a relatively minor influence on the adsorption of reactants. It significantly enhances the material’s electrical conductivity, thereby improving the efficiency of electrocatalytic processes [61]. Finally, the introduction of oxidized nitrogen enhances the surface hydrophilicity, further optimizing catalytic performance. By strategically adjusting the distribution of different nitrogen species, the electrocatalytic performance of the cathode in zinc–air batteries can be effectively improved, thereby enhancing their stability and extending their cycle life [56]. Controlling the doping sites and the proportions of nitrogen species can further improve their electrical conductivity, active site density, and ion storage performance. This strategy is also a key direction in current research on bio-based carbon aerogels due to its wide applicability and strong development potential.

#### 3.1.2. In Situ Doping

The in situ doping strategy involves the incorporation of nitrogen-containing components (such as urea, melamine, amino acids, or inherently nitrogen-rich biomasses) into the biomass precursors prior to their carbonization [64]. During the subsequent pyrolysis process, this nitrogen source decomposes while the carbon framework is simultaneously reconstructed, enabling nitrogen atoms to be embedded into the formed carbon lattice. This parallel process ensures the homogeneous distribution of nitrogen species, exceptional structural stability, and strong coupling with carbon nucleation centers [65]. At the microscopic level, this approach can regulate the local electron density and bonding environment, and promote the formation of defect sites, thereby enabling precise control of the types and proportions of active centers, such as graphitic N and pyridinic N. Consequently, it is considered one of the fundamental methods for preparing high-performance nitrogen-doped bio-based carbon aerogels with stable structural and electrochemical active sites [66]. Pyrolysis temperature also plays a critical role in determining the nitrogen content and nitrogen configuration of the resulting carbon framework. As the temperature increases, the fractions of pyrrolic N and pyridinic N, which possess lower thermal stability, tend to decrease, while the proportion of graphitic N, which exhibits higher thermal stability, correspondingly increases. Meanwhile, a higher pyrolysis temperature generally promotes the carbonization process and enhances electrical conductivity, yet an excessively high temperature causes substantial nitrogen loss and alters the pore structure. Therefore, the influence of pyrolysis temperature should be considered from the perspective of an overall balance among nitrogen retention, nitrogen configuration, electrical conductivity, and pore structure [67]. Wei et al. reported a bio-based nitrogen-doped carbon aerogel (N-CA) based on nanocellulose (CNF) and g-C_3_N_4_ for the air cathode of a zinc–air battery [68]. During the hydrothermal pretreatment process, the nitrogen-rich g-C_3_N_4_ combined with the CNF precursor, and subsequent carbonization achieved in situ nitrogen doping, primarily forming graphitized and pyridinic N species that effectively modulate the electronic structure and enhance oxygen reduction and oxygen evolution reaction activity. The CNF skeleton provided a continuous three-dimensional porous network with a high specific surface area of 785.4 m^2^ g^−1^ and efficient mass transport pathways. N1s analysis showed that graphitic and pyridinic N made up 69.1% of the total, which helped achieve excellent bifunctional electrocatalytic performance. Zinc–air batteries assembled with this carbon aerogel achieved a specific capacity of 790 mAh g^−1^, an energy density of 945.6 Wh kg^−1^, and remained stable for over 100 h of operation, demonstrating the potential of in situ nitrogen-doped carbon aerogels for efficient air cathodes (Table 2).

**Figure 6:**
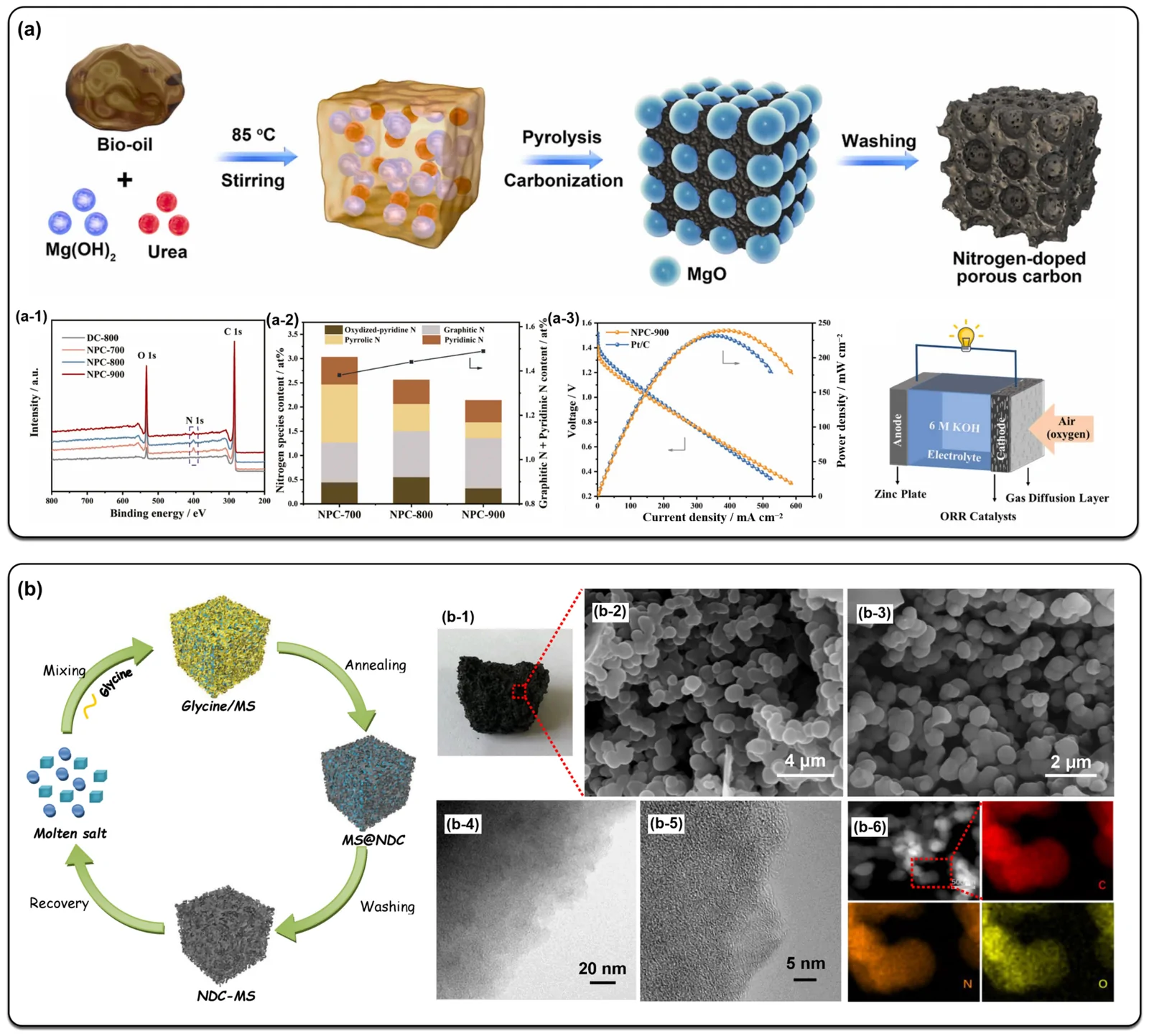
(**a**) Schematic illustration of the synthesis of NPC-T [69]. Copyright 2023, Elsevier. (**b**) Schematic illustration for synthesizing NDC-MS via the molten salt method [70]. Copyright 2020, Elsevier.

Xu et al. prepared nitrogen-doped hierarchical porous carbon using bio-oil as the carbon source and urea as the nitrogen source via a one-step MgO-templated high-temperature pyrolysis method [69] (Figure 6a). During carbonization, urea undergoes in situ decomposition, which incorporates nitrogen elements into the carbon framework and generates abundant active sites of graphitic nitrogen and pyridinic nitrogen. The obtained material combined a hierarchical pore structure with a high nitrogen content, which collectively enhanced its ORR activity (half-wave potential of 0.84 V). When assembled into a zinc–air battery, the cathode achieved a high peak power density of 239.36 mW cm^−2^ and exhibited excellent discharge stability (Table 2). Cai et al. developed a metal-free, nitrogen-doped carbon aerogel for zinc–air batteries using glycine as both the carbon and nitrogen source via a molten salt templating method (Figure 6b) [70]. The resulting aerogel featured an interconnected 3D hierarchical porous network with a specific surface area of up to 1548.6 m^2^ g^−1^, which facilitated the exposure of active sites and the rapid transmission of electrolytes. This architecture conferred outstanding ORR performance, with a half-wave potential of 0.88 V for the oxygen reduction reaction and a nearly 4-electron transfer pathway, indicating excellent catalytic activity and reaction kinetics. The corresponding zinc–air battery exhibited an open-circuit voltage of 1.438 V, a peak power density of 174 mW cm^−2^, a specific capacity of 753 mAh g^−1^, and stable operation over 72 h of cycling (Table 2). These results indicated that in situ nitrogen doping is an extremely effective strategy for creating efficient and durable air cathodes with significant potential for sustainable electrocatalytic applications.

In situ doping facilitates the incorporation of heteroatoms into the carbon framework during carbonization, which generally leads to a more uniform distribution of doped species. However, the formation of specific heteroatom configurations is difficult to precisely control because it is influenced by the precursor structure, doping source, and carbonization conditions. Therefore, further optimization of precursor design and thermal treatment processes is required to regulate the type and concentration of active sites.

#### 3.1.3. Post-Treatment Doping

The post-treatment doping strategy involves first synthesizing the carbon material, followed by the introduction of nitrogen atoms through methods such as ammonia heat treatment, nitrogen source vapor infiltration, or wet-chemical modification [71]. The principal advantage of this approach is that it can selectively construct new nitrogen functional groups on the surface and defect regions of the material while maintaining the integrity of the original carbon framework. This method is particularly suitable for increasing the density of surface-active sites, enhancing the efficiency of catalytic interfacial reactions, and adjusting the wettability of the material surface. Furthermore, post-treatment can typically introduce more diverse surface functional groups than in situ methods [72], which can significantly improve electrochemical interfacial reactions, adsorption capacities, and electrolyte compatibility. Given its versatility and precision in surface engineering, post-treatment doping has become a pivotal technological route for constructing nitrogen-doped carbon materials with high-activity surfaces. Compared with in situ doping, post-treatment doping provides greater flexibility in modifying surface nitrogen species and exposed active sites, whereas in situ doping generally promotes a more uniform incorporation of dopants within the carbon framework. In situ doping also integrates dopant incorporation with carbonization, which simplifies the synthesis process and is favorable for scale-up. Post-treatment doping requires an additional treatment step but allows the surface chemical environment to be adjusted after the carbon framework has been formed. These differences influence not only the distribution of nitrogen species, but also the preservation of the porous framework and the accessibility of surface-active sites [73].

**Figure 7:**
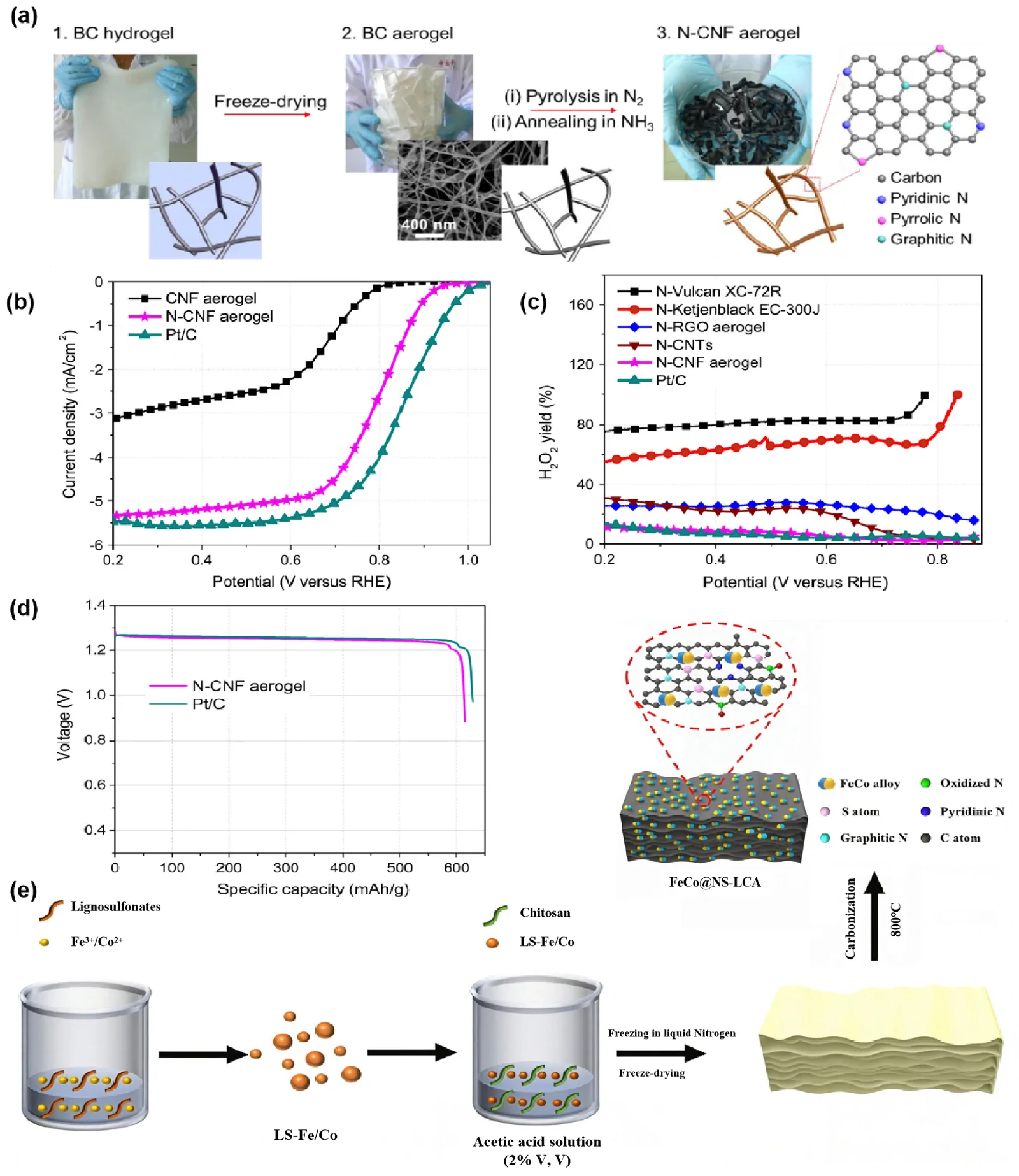
(**a**) Schematic diagram of the synthetic steps. (**b**) ORR polarization plots of CNF aerogel, N-CNF aerogel, and Pt/C catalyst. (**c**) H2O2 yield plots of N-CNF aerogel and reference catalysts. (**d**) Long-term galvanostatic discharge curves of Zn-air batteries until complete consumption of the Zn anode. The specific capacity was normalized to the mass of consumed Zn [74]. Copyright 2015, Elsevier. (**e**) Schematic illustration of the synthesis process of FeCo@NS-LCA [75]. Copyright 2024, Elsevier.

Liang et al. developed a nitrogen-doped carbon nanofiber aerogel (N-CNF) derived from bacterial cellulose [74]. Post-processing nitrogen doping was achieved via secondary heat treatment of the pre-formed bacterial cellulose-based carbon network under an NH_3_ atmosphere (Figure 7a). This NH_3_ treatment effectively introduced abundant pyridinic and graphitic nitrogen active sites on the carbon framework. Crucially, this process retained the three-dimensional nanofiber network and a high surface area of 916 m^2^ g^−1^, ensuring exposure of numerous active sites and rapid mass-transmission pathways. Consequently, the N-CNF aerogel exhibited excellent ORR performance, characterized by a half-wave potential of 0.80 V (Figure 7b) and an H_2_O_2_ yield below 10% (Figure 7c), which indicates its excellent four-electron selectivity and reaction kinetics. When employed as an air cathode in a zinc–air battery, the N-CNF aerogel delivered a specific capacity of 615 mAh g^−1^ (Figure 7d) and an energy density of 760 Wh kg^−1^, demonstrating its capability to effectively replace the commercial Pt/C catalyst (Table 2). These results indicate that post-treatment nitrogen doping is an effective method for developing high-performance and durable BC-derived N-CNF aerogels for advanced air cathodes.

Post-treatment doping mainly modifies the surface chemistry of preformed carbon frameworks. This approach allows additional functionalization after pore formation, which is beneficial for adjusting surface defects and heteroatom species. Nevertheless, harsh oxidation or chemical treatment may alter the pore structure or introduce excessive defects, making the selection of treatment conditions important.

#### 3.1.4. Co-Doping Strategy

The co-doping strategy involves the simultaneous introduction of nitrogen and other heteroatoms (such as sulfur (S), phosphorus (P), or boron (B)) into the carbon framework. This approach exploits the synergistic interactions among multiple elements to regulate the electronic structure of the carbon framework, thereby optimizing its band structure, enhancing material polarity, and cooperatively improving the activity of catalytic sites [76]. The differences in electronegativity and atomic radius among these doped heteroatoms lead to significant charge heterogeneity within the carbon network, thereby promoting the adsorption of reactants, facilitating electron transfer, and enhancing interfacial catalytic kinetics [77]. For instance, the co-doping of N and S can create a donor-acceptor electronic configuration, while the co-doping of N and P can effectively adjust the distribution of π electrons and enhance the structural stability [78]. Consequently, the co-doping strategy has emerged as a prominent research direction for improving the catalytic and energy storage performance of bio-based carbon aerogels in recent years.

Lignin has attracted considerable interest in the field of co-doped carbon materials due to its abundant aromatic structures and oxygen-containing functional groups. The oxygen-rich groups in lignosulfonate can chelate metal ions, which prevents the agglomeration of metal nanoparticles during carbonization and catalytic processes. Han et al. constructed a N, S co-doped carbon aerogel (FeCo@NS-LCA) loaded with FeCo alloy using lignosulfonate and chitosan as biomass precursors [75]. The three-dimensional hierarchical porous structure formed by directional freeze-drying was conducive to the uniform introduction of heteroatoms and the exposure of active sites. Co-doping N and S in the carbon skeleton synergistically regulated the electronic structure and enhanced the adsorption and activation ability of oxygen molecules (Figure 7e), enabling the half-wave potential of the ORR of this material to reach 0.87 V, with the overpotential of the OER being only 324 mV. The assembled Zn-air battery delivered a specific capacity of 764 mAh g^−1^ at 10 mA cm^−2^ and maintained stable cycling for over 1500 h. Ravichandran et al. developed a supramolecular modulation strategy, in which lignin was used as the precursor and uniformly dispersed in a Lewis acid-base supramolecular system [79]. After high-temperature pyrolysis, N, P co-doped carbon material with topological defects was obtained, which was named LNPC-1000. This material exhibited ORR onset and half-wave potentials of 0.97 V and 0.85 V, respectively. After a chronoamperometric test lasting 15,000 s, the current retention remained at 95.4% of its initial value, demonstrating superior stability and resistance to methanol crossover compared to commercial Pt/C. Furthermore, at a current density of 10 mA cm^−2^, the overpotential for the OER was 480 mV. The Zn-air battery assembled with LNPC-1000 delivered an open-circuit voltage of 1.47 V and a peak power density of 143.5 mW cm^−2^. At a current density of 10 mA cm^−2^, its specific capacity reached 784.6 mAh g^−1^, and its charge–discharge cycling stability could be maintained for more than 1200 h. (Table 2).

**Figure 8:**
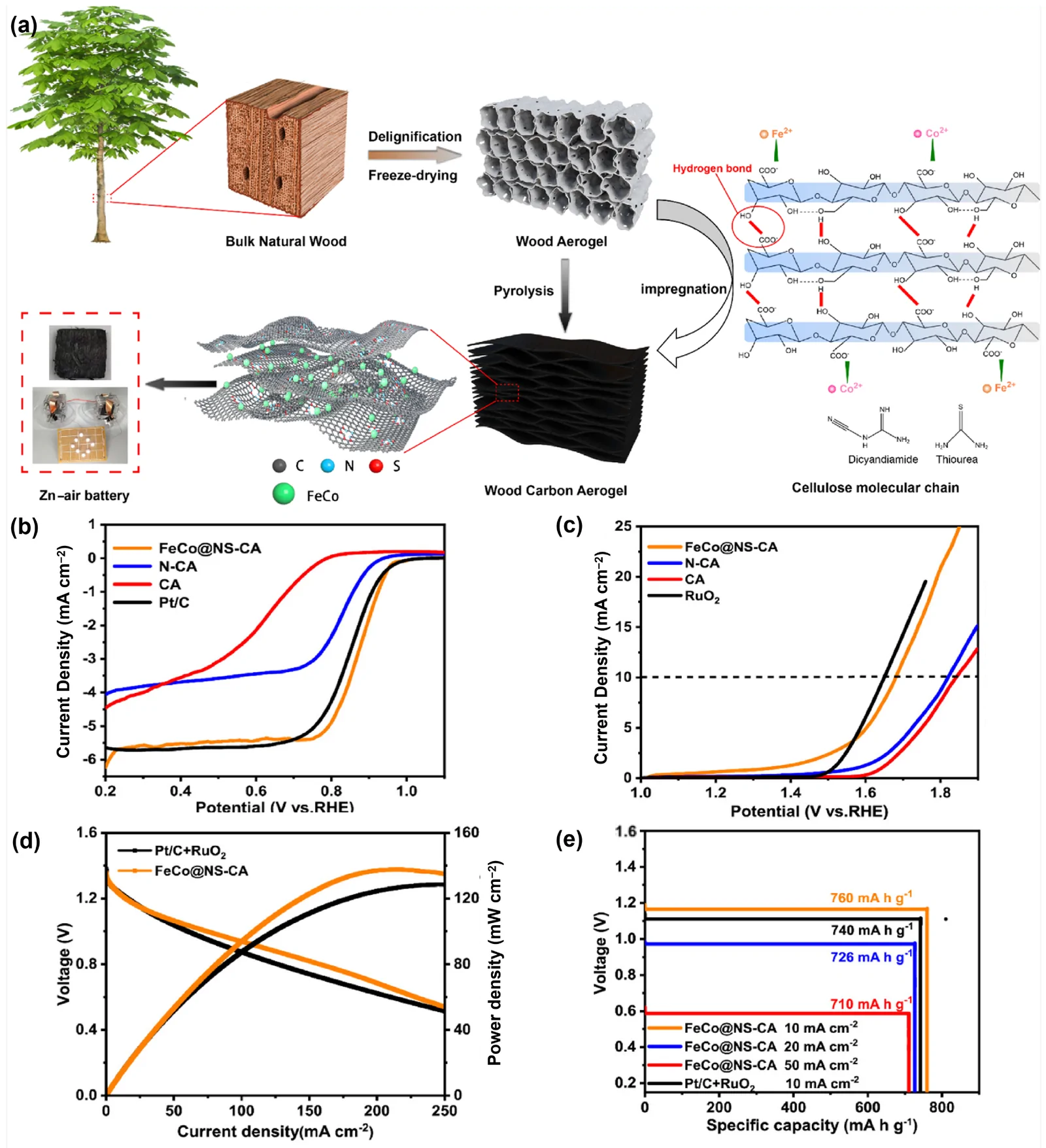
(**a**) Schematic diagram of the synthesis process of FeCo@NS-CA. (**b**) ORR polarization curves. (**c**) OER polarization curves with iR correction. (**d**) Polarization and power density curves of ZABs with FeCo@NS-CA and Pt/C + RuO2. (**e**) Specific capacity curves of ZABs with FeCo@NS-CA and Pt/C + RuO2 [80]. Copyright 2021, ACS.

Pang et al. employed delignified balsa wood as the biomass precursor to prepare a N,S co-doped hierarchical porous carbon aerogel (FeCo@NS-CA) loaded with FeCo alloy as a bifunctional electrocatalyst for the cathodes of zinc–air batteries [80] (Figure 8a). The natural three-dimensional porous framework derived from the wood layer structure was obtained in this material, and efficient co-doping of N and S heteroatoms was achieved, which is beneficial for regulating the electronic structure of the carbon-based electrocatalysts and increasing the density of active sites. The obtained FeCo@NS-CA demonstrated excellent electrocatalytic performance in alkaline media, with an ORR onset potential of 0.97 V and a half-wave potential of 0.85 V, comparable to that of commercial Pt/C (Figure 8b). Furthermore, the material exhibited a competitive OER overpotential of 450 mV, similar to that of the benchmark RuO_2_ catalyst (Figure 8c), indicating its excellent bifunctional activity. When FeCo@NS-CA was employed as the air cathode in a zinc–air battery, the device achieved a peak power density of 140 mW cm^−2^ (Figure 8d) and a specific capacity of 760 mAh g^−1^ at 10 mA cm^−2^ (Figure 8e). The battery also showed remarkable long-term stability, enabling stable cycling for over 400 h with performance significantly superior to that of the Pt/C + RuO_2_ counterpart (Table 2). These studies collectively demonstrate the potential of bio-based co-doped carbon aerogels as high-performance, durable electrocatalysts for advanced zinc–air batteries.

**Table 2:** Summarizes the performance parameters of the nitrogen-doped and co-doped carbon aerogels.

Strategy	S_BET_ (m^2^ g^−1^)	D avg (nm)	E_1/2_ (V)	η (mV)	Pmax (mW cm^−2^)	Stability (h)	Ref.
In situ N	785.4	-	0.86	410	270	100	[68]
In situ N	1218	3.58	0.84	-	239	24	[69]
In situ N	1548.6	0.7–22 (PSD)	0.88	-	174	72	[70]
Post-treatment N	916	-	0.80	-	-	-	[74]
N, S + FeCo	621.61	7.34	0.87	324	-	1500	[75]
N, P co-doping	903.1	2.6	0.85	480	144	1200	[79]
N, S + FeCo	164	3–5 (PSD)	0.85	450	140	400	[80]
N, B co-doping	549.02	4	0.85	310	371	300	[81]
N, S + FeCo	123.6	3–4 (PSD)	0.85	335	132	250	[82]
N, S co-doping	988.17	6	0.85	-	151	-	[83]
N + Fe	544.41	6.73	0.84	250	125	147	[84]
In situ N	745.12	-	0.86	-	249	300	[85]
N + FeS	176.67	4	-	322	176	48	[86]
In situ N	898	3.4	0.86	-	186	-	[87]
In situ N	587.8	-	0.89	-	206	400	[88]
N + Co	306.57	20–50 (PSD)	0.85	-	155	157	[89]

Pmax = peak power density. “-” = not reported. E_1/2_ = Half-wave potential. SAs = Single atoms. η = OER overpotential. D avg = average pore diameter. PSD = Pore size distribution.

In addition to N, S and N, P co-doping, N, B co-doping is also used to regulate the electronic structure of carbon-based catalysts. Zhang et al. prepared a wood-derived N, B co-doped carbon catalyst (Co@CoO/BNC) [81]. The B-N-C moieties formed by co-doping synergistically modulated the charge distribution and induced abundant topological defects. The synergistic effect between B and N heteroatoms enhanced the adsorption and activation of oxygen intermediates and achieved an ORR half-wave potential of 850 mV and an OER overpotential of 310 mV at 10 mA cm^−2^. The assembled zinc–air battery delivered a specific capacity of 958.5 mAh g^−1^ and cycled stably for over 300 h (Table 2). The synergistic effect of co-doping originates from the interaction between different heteroatoms rather than the simple accumulation of multiple elements. For example, the combination of N with S or P can modify local charge distribution and regulate intermediate adsorption behavior. However, the catalytic contribution of each dopant is highly dependent on its bonding configuration and spatial arrangement within the carbon matrix, which requires further clarification.

Table 2 summarizes the representative electrochemical and zinc–air battery performance reported for different nitrogen-doping and co-doping strategies. In situ N doping generally provides relatively consistent ORR activity, with E_1/2_ values of 0.84–0.89 V and peak power densities of 174–270 mW cm^−2^ among the representative examples. This strategy enables nitrogen incorporation during precursor synthesis or carbonization, facilitating the formation of N-containing active sites within the carbon framework. Post-treatment N doping provides an alternative route for modifying the surface composition after carbonization. For example, the sample reported in exhibits a relatively high specific surface area of 916 m^2^ g^−1^, but its E_1/2_ is 0.80 V, indicating that a high specific surface area alone does not necessarily lead to enhanced ORR activity [74]. Compared with single N doping, co-doping with heteroatoms or transition metals provides additional opportunities to regulate the electronic structure and active-site environment. Some co-doped systems exhibit improved battery performance, such as the N, B co-doped catalyst with a Pmax of 371 mW cm^−2^ and the N, S + FeCo co-doped catalyst with a stability of 1500 h [75,81]. However, the performance varies considerably among different co-doped systems, suggesting that the effectiveness of co-doping depends on the dopant combination and the resulting active-site configuration. These values provide a useful overview of the reported performance, but differences in catalyst loading, electrolyte concentration, electrode structure, current density, and cell configuration should also be considered when comparing the results. In particular, the ORR half-wave potential and OER overpotential are primarily associated with the electrocatalytic activity, whereas peak power density and cycling stability are also affected by the overall battery configuration and operating conditions.

The Zn-air battery is a coupled system in which the Zn anode, electrolyte, separator, gas diffusion layer, and gas–liquid-solid interface all contribute to the overall lifetime. Therefore, long-term cycling alone cannot establish the intrinsic robustness of the carbon aerogel catalyst. Intrinsic catalyst durability is better assessed in half-cell configurations, such as rotating disk electrode/rotating ring-disk electrode (RDE/RRDE) measurements, by accelerated durability tests, chronoamperometry, and repetitive ORR/OER potential cycling, using metrics including shifts in half-wave potential and onset potential, limiting-current retention, and ECSA loss, together with post-test XPS, XAS, and AC-HAADF-STEM analysis.

The degradation of zinc–air batteries is a combination of several coupled mechanisms happening simultaneously at the cell and catalyst levels. At the cathode, carbon corrosion occurs at high charging potentials, leading to pore structure collapse, reduced electrical conductivity, and detachment of active sites, which is more significant in defect-rich, high specific surface area aerogels. Alkaline media can lead to dissolution or leaching of metal species in M-N-C sites or metal nanoparticles, especially at high current density and extended cycling, resulting in a permanent loss of active centers. In addition, the M-N-C sites can be protonated, oxidized, demetalated, and coordination rearranged during ORR/OER, leading to formation of less active or inactive phases, such as metal oxides/hydroxides. At the cell level, alkaline electrolytes readily absorb CO_2_ from air to form carbonate/bicarbonate species, which reduces ionic conductivity, alters local pH, blocks pores, and increases interfacial resistance. The Zn anode can also suffer from passivation and dendrite growth through insulating ZnO or zincate layers, resulting in shape change and poor reversibility. In addition, an imbalance at the gas–liquid-solid three-phase interface may cause flooding that blocks O_2_ diffusion or drying that interrupts ionic pathways, both of which accelerate performance decay. Because these processes are often intertwined, distinguishing their individual contributions requires operando/in situ spectroscopy, post-mortem analysis, and control experiments such as symmetric cells, separate anode/cathode tests, and EIS [90].

### 3.2. Non-Noble Metal-Doped Bio-Based Carbon Aerogels

Besides the strategy of doping with heteroatoms, the incorporation of non-noble metals into the bio-based carbon carriers is another key approach for enhancing the cathode performance of zinc–air batteries [4]. Specific pore parameters strictly dictate the viability and accessibility of different active sites. For Single-Atom Catalysts (SACs) featuring M-N_4_ coordination, an abundance of intrinsic micropores (<2 nm) is critical. Micropores provide a high density of topological defects and edge sites that act as spatial traps to anchor isolated metal atoms, effectively preventing their migration and agglomeration during high-temperature pyrolysis. Conversely, for the growth of metal nanoparticles, a well-developed network of mesopores (2–50 nm) is mandatory. Mesopores physically accommodate the larger dimensions of nanoparticles, preventing the severe pore blockage that would otherwise occur in purely microporous matrices, while simultaneously providing broad channels for the rapid mass transport of bulky oxygen intermediates and electrolyte ions [91]. Biomass carbons typically possess hierarchical pore structures, high specific surface areas, and excellent electrical conductivity, which enable them to effectively stabilize various non-noble-metal active centers, including metal/metal-oxide nanoparticles [92], metal clusters [93], and metal single atoms (SACs) [94]. These metal centers can accelerate oxygen electrocatalysis by forming M-C, M-O, M-N, M-S, or M-P bonds, as well as through defect-anchored structures [95]. When dispersed on biomass carbon supports, typical non-noble metals such as Co, Fe, and Mn can significantly increase the ORR half-wave potential, decrease the OER overpotential, and narrow the charge–discharge voltage gap, thereby enabling ZABs with higher power density, larger specific capacity, and better cycling durability. Notably, nitrogen doping often serves as a crucial regulatory factor for further enhancing catalytic efficiency beyond the intrinsic activity of metal centers alone. Nitrogen atoms can modify the electronic structure, strengthen the anchoring of metal sites, and form M-N_x_ coordination configurations, which collectively improve the utilization efficiency and intrinsic activity of metal active centers. In summary, nitrogen-modified non-noble metal-doped carbons (M-N-C) exhibit stronger interfacial synergistic effects and more outstanding bifunctional catalytic performance. This synergy underscores an important development pathway for constructing high-performance ZAB cathode materials [96,97].

#### 3.2.1. Metal Particle-Doped Carbon Aerogels

**Figure 9:**
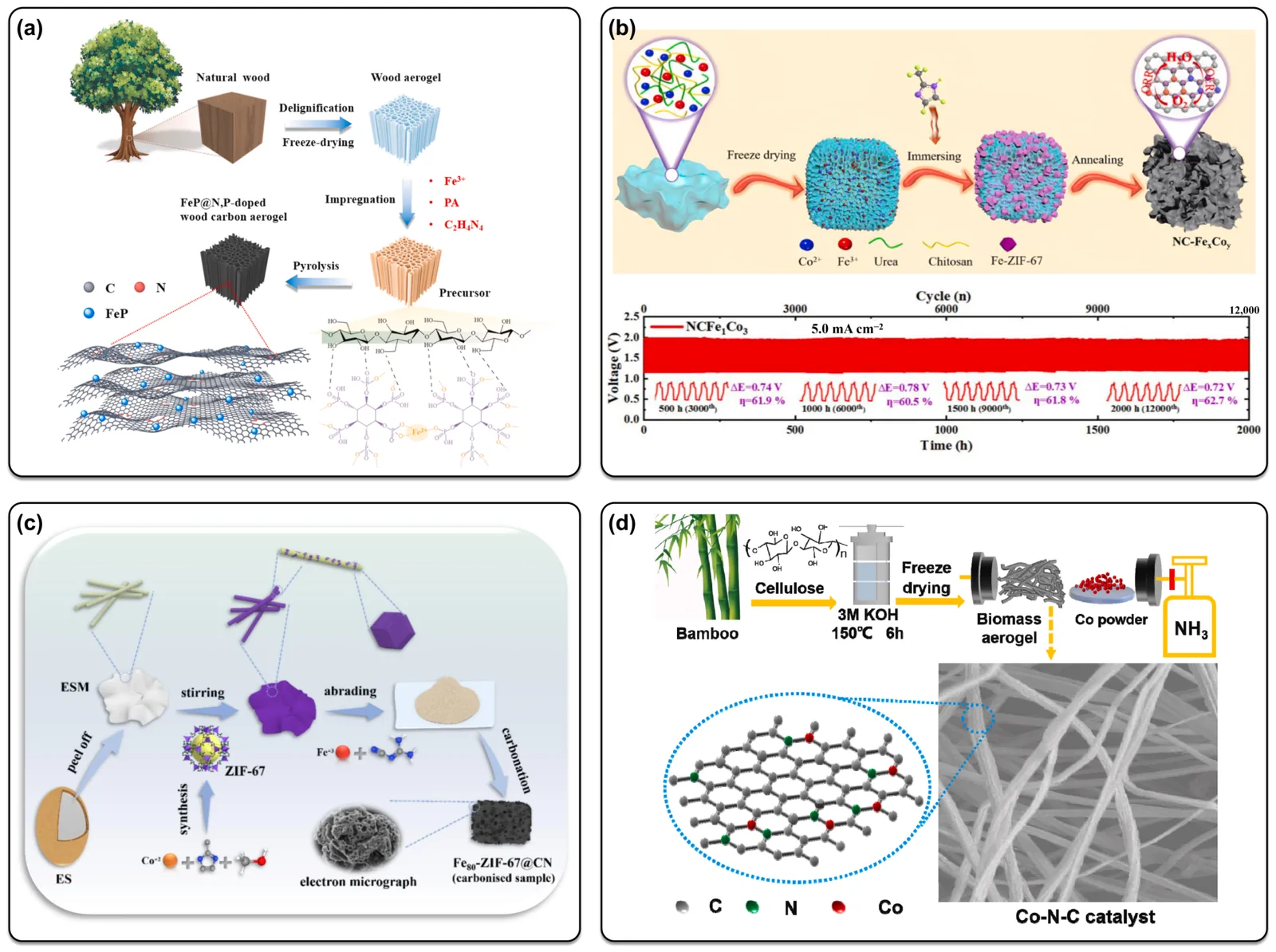
(**a**) Schematic diagram of the preparation process of FeP@N,P-WCA [98]. Copyright 2024, Elsevier. (**b**) Schematic diagram of the formation process [100]. Copyright 2025, Elsevier. (**c**) Synthetic strategy of Fe80-ZIF-67@CN [101]. Copyright 2024, Elsevier. (**d**) Schematic illustration of the synthesis of the Co-N-C single-atom catalyst [102]. Copyright 2025, Elsevier.

Metal-loaded carbon materials serve as attractive electrocatalyst supports, where their porous and conductive carbon matrix can effectively stabilize the metal species, enhance electron transfer, and facilitate gas diffusion. For instance, Xu et al. designed a wood-derived nitrogen-phosphorus co-doped carbon aerogel containing uniformly distributed iron phosphide nanoparticles (FeP@N,P-WCA) (Figure 9a) [98]. The synergistic interaction among Fe, N, and P regulated the electronic structure of the carbon framework and improved the accessibility of catalytic sites. The catalyst exhibited an onset potential of 0.95 V, a limiting current density of 5.20 mA cm^−2^, and an ORR half-wave potential of 0.84 V. When assembled into a zinc–air battery, it achieved an open-circuit voltage of 1.464 V and a specific capacity of 775.5 mAh g^−1^ at 5 mA cm^−2^, with stable cycling performance over 160 h (Table 3). Qian et al. fabricated Fe, N co-doped porous carbon-based electrocatalysts with a three-dimensional porous structure using industrial sodium lignosulfonate as a precursor [99]. The abundant phenolic hydroxyl and carboxyl groups in lignin ensured the uniform dispersion of Fe-N_x_ active sites. In alkaline media, the half-wave potential of the as-prepared catalyst reached 0.90 V, and the catalyst simultaneously exhibited excellent methanol tolerance and long-term cycling stability. The Zn-air battery assembled with this catalyst delivered an open-circuit voltage of 1.477 V and a peak power density of 125 mW cm^−2^ (Table 3).

Similarly, Zhou et al. fabricated nitrogen-doped carbon aerogels embedded with Fe-Co nanoalloys (NC-FeCo) via a chitosan precursor, which was applied as a high-performance air cathode for rechargeable zinc–air batteries (ZABs) (Figure 9b) [100]. The coupling between FeCo nanoparticles and the N-doped carbon matrix generated multiple active sites, including M-N_x_, pyridinic N, and graphitic N, which contributed to oxygen intermediate conversion. The catalyst exhibited an ORR onset potential of 1.01 V, a half-wave potential of 0.88 V, and an OER overpotential of 378 mV at 10 mA cm^−2^. When applied as an air cathode, NC-FeCo delivered a peak power density of 240 mW cm^−2^ and maintained stable operation for over 2000 h at 5 mA cm^−2^ (Table 3). In addition, Yang et al. employed an eggshell membrane as a biomass-derived precursor to fabricate a CoFe alloy nanoparticle-embedded N-doped carbon material (Fe_80_-ZIF-67@CN) through a ZIF-67 templating route followed by high-temperature carbonization (Figure 9c) [101]. The coexistence of CoFe nanoparticles and Fe-N_x_/Co-N_x_ sites provided coupled catalytic centers for ORR and OER. The catalyst achieved an ORR half-wave potential of 0.86 V and an OER overpotential of 339 mV at 10 mA cm^−2^. The assembled zinc–air battery exhibited an open-circuit voltage of 1.54 V, a peak power density of 126.47 mW cm^−2^, a specific capacity of 773.63 mAh g^−1^, and stable cycling performance over 176 h (Table 3).

Overall, metal nanoparticles provide abundant catalytic interfaces for oxygen electrocatalysis, while the surrounding heteroatom-doped carbon matrix plays an important role in regulating electronic structures and stabilizing metal species. However, maintaining the dispersion and structural stability of metal nanoparticles during long-term operation remains an important consideration for practical applications.

#### 3.2.2. Metal Single-Atom Doped Carbon Aerogels

Compared with metal nanoparticles, SACs feature ultrahigh atomic utilization, uniformly and atomically identifiable active sites, and excellent thermal and chemical stability. These characteristics endow SACs with superior mass activity and enhanced durability in ORR/OER bifunctional catalysis [94]. Despite these advantages, the identification of genuine single-atom sites remains critical. Sub-nanometer clusters or very small metal nanoparticles may be difficult to distinguish from isolated metal atoms using conventional characterization methods [103]. Therefore, the assignment of isolated metal centers should be supported by complementary evidence from multiple characterization techniques rather than a single measurement. Aberration-corrected high-angle annular dark-field scanning transmission electron microscopy (HAADF-STEM) can provide direct evidence for the spatial dispersion of metal species, while X-ray absorption near-edge structure/extended X-ray absorption fine structure (XANES/EXAFS) and X-ray photoelectron spectroscopy (XPS) provide complementary information on their local coordination environments and chemical states [104]. For Fe-based catalysts, Mössbauer spectroscopy can further probe the local Fe environment [105], whereas theoretical calculations can help evaluate the plausibility of the proposed metal–nitrogen (M-N_x_) configurations. Under ORR/OER conditions, operando X-ray absorption spectroscopy (operando XAS) is particularly valuable for monitoring changes in the coordination environment and possible migration or aggregation of metal species during electrochemical operation [104]. Mechanistically, the hierarchical pores of bio-based carbon aerogels offer sufficient space for the formation and stabilization of isolated metal centers, while their abundant surface functionalities further regulate the electronic structures surrounding these centers. By inducing local charge redistribution at M-N_x_ coordination sites and altering the adsorption energetics of O_2_ and key intermediates, these carbon-based matrices significantly modify reaction pathways and accelerate overall oxygen-electrocatalysis kinetics. In recent years, biomass-derived carbon-based single-atom catalysts (SACs) have demonstrated remarkable potential as air-cathode materials for zinc–air batteries. In these systems, isolated transition-metal atoms such as Fe, Co, and Ni are anchored on biomass-derived carbon frameworks, which are typically nitrogen-rich or co-doped with other heteroatoms, thereby forming well-defined M-N_x_ single-atom active sites [4]. The synthesis of these advanced materials typically relies on strategies that include molecular-level precursor mixing followed by high-temperature carbonization [106], encapsulation-carbonization routes using MOF/biomass-precursor [107], and in situ reduction assisted by plasma or wet chemistry [108].

##### N-Coordinated Single-Atom Catalysts (N-Coordinated SACs)

Driven by the abundant natural reserves of nitrogen in biological precursors, nitrogen-coordinated single-atom catalysts represent the most fundamental and extensively investigated structure in biomass-based carbon aerogels. The classic symmetric metal-N_4_ (M-N_4_) configuration can effectively anchor isolated transition metal atoms to prevent their agglomeration during high-temperature carbonization and simultaneously act as the active center for the high-performance oxygen reduction reaction (ORR). In addition to conventional isolated single-atom sites, advanced structural engineering strategies have recently enabled the construction of a complex, high-density quasi-planar Fe_2_N_6_ configuration, which provides an enhanced synergistic catalytic pathway for efficient oxygen reduction reactions. These engineered nitrogen-coordination environments maximize atomic utilization and significantly accelerate the kinetics of the bifunctional electrocatalytic reactions required for air cathodes.

**Figure 10:**
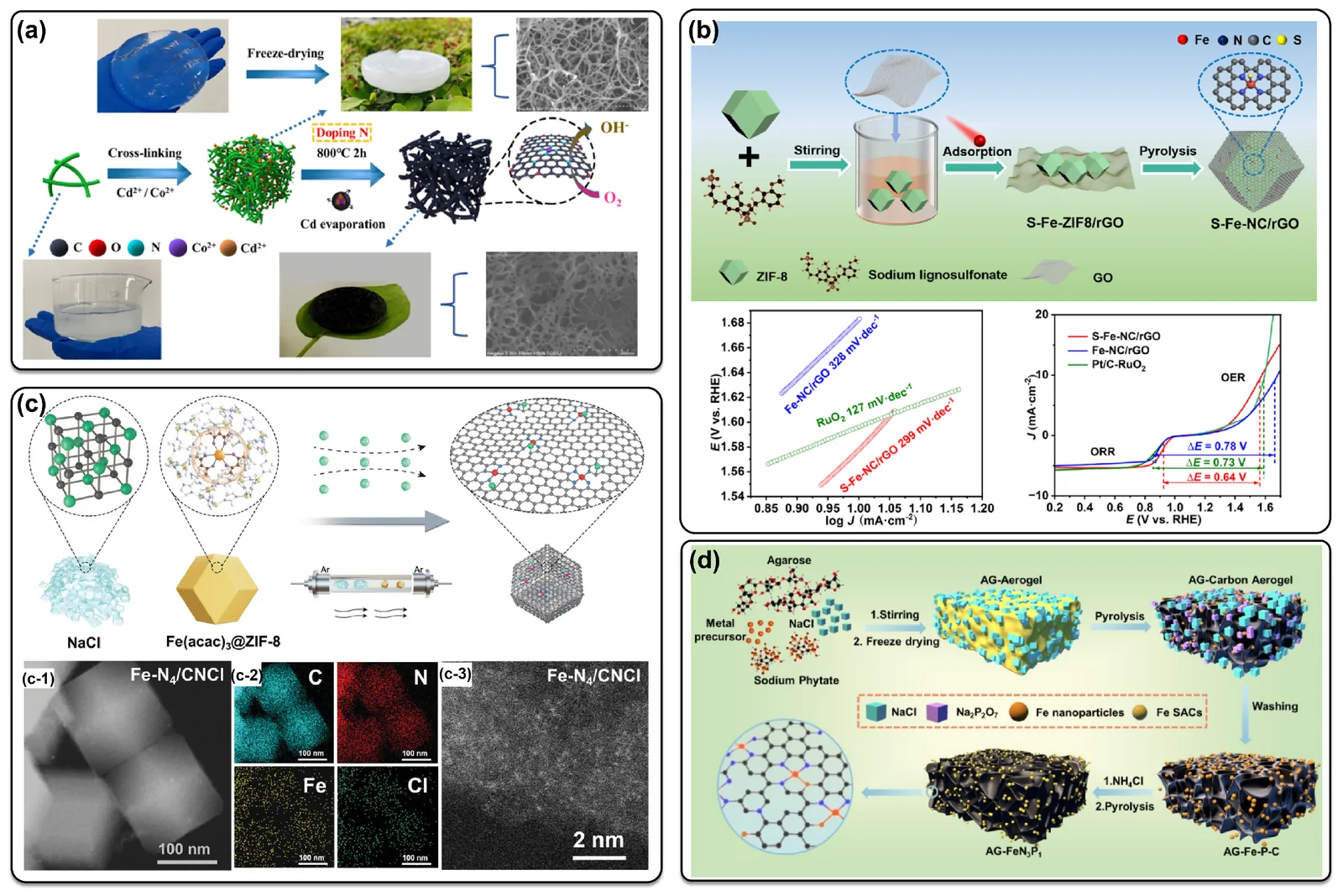
(**a**) Schematic diagram for the preparation of Co SAs/NCNA [109]. Copyright 2023, Elsevier. (**b**) Synthesis of S-Fe-NC/rGO [110]. Copyright 2024, ACS. (**c**) The schematic illustration of the synthetic procedure of the Fe-N4/CNCl catalyst [111]. Copyright 2024, John Wiley and Sons. (**d**) Schematic diagram of the preparation of AG-FeN3P1 SACs [112]. Copyright 2025, Elsevier.

Yang et al. fabricated a bamboo-derived biomass carbon aerogel catalyst (Co-N-C-1050), in which cobalt atoms are dispersed at the atomic level in the form of Co-N_4_ sites, endowing the material with excellent bifunctional catalytic activity (Figure 9d) [102]. It exhibited an ORR half-wave potential of 0.842 V, an electron transfer number close to 4, and an OER potential of 1.472 V at 10 mA cm^−2^, outperforming the N-C-1050, Pt/C, and RuO_2_ benchmarks. Upon integration into a zinc–air battery, it delivered an open-circuit voltage of 1.49 V and a peak power density of 135 mW cm^−2^. The battery showed negligible voltage decay after 138 h of charge–discharge cycling, demonstrating that single-atom Co significantly enhanced bifunctional electrocatalytic activity and cycling stability (Table 3). Shen et al. constructed a TOCNFs-Cd^2+^/Co^2+^ coordinated aerogel precursor using wood-derived TEMPO-oxidized cellulose nanofibers (TOCNFs), which provide abundant metal-anchoring sites (Figure 10a) [109]. This precursor facilitated the uniform diffusion of cobalt atoms, successfully resulting in cobalt single-atom-anchored N-doped carbon nanofiber aerogels (Co SAs/NCNA). During this process, Cd (boiling point 767 °C) acted as a low-boiling thermal sacrificial agent, evaporating during carbonization and generating abundant micropores. Spatial limitation effectively inhibited Co aggregation and stabilized the formation of Co-N_4_ single-atom structures. The obtained material exhibited a high specific surface area of 2265.1 m^2^ g^−1^ and a pore volume of 2.24 cm^3^ g^−1^, forming a hierarchical porous structure composed of micro-, meso-, and macropores. Benefiting from abundant single-atom active sites, rapid gas/ion transport channels, and high electrical conductivity, Co SAs/NCNA exhibited an ORR half-wave potential of 0.855 V in alkaline media and possessed excellent cycling durability. When applied to a zinc–air battery, it could deliver an open-circuit voltage of 1.49 V, a specific capacity of 769 mAh g^−1^, and a peak power density of 206 mW cm^−2^, with all these key metrics surpassing those of the conventional Pt/C + RuO_2_ benchmark (Table 3). These results clearly demonstrated the high catalytic efficiency and application potential of this biomass-derived cobalt single-atom catalyst in zinc–air batteries.

##### N/S Coordinated Single-Atom Catalysts (N/S Coordinated SACs)

Simultaneous introduction of sulfur and nitrogen is employed to construct nitrogen/sulfur co-coordinated single-atom catalysts (SACs). This method leverages the larger atomic radius and unique electronegativity of sulfur to induce favorable lattice strain. Using specific biomass derivatives (e.g., 2,5-diformylfuran), researchers have successfully constructed asymmetric coordination structures such as Co_1_-N_3_S_1_. The introduction of sulfur into the first coordination shell disrupts the symmetric electron distribution of conventional metal-nitrogen sites, which upshifts the d-band center of the metal and brings it closer to the Fermi level. This directional electron redistribution accelerates the sluggish reaction kinetics, thereby achieving excellent bifunctional catalytic activity within the three-dimensional porous network of aerogels. Such asymmetric coordination can also affect the adsorption of key oxygen intermediates, including *OOH, *O, and *OH, by changing their interaction with the metal center. First, the larger atomic radius and lower electronegativity of S, when introduced into the first coordination shell of an M-N_4_ moiety, break the in-plane symmetry and create an asymmetric ligand field. This geometric distortion, together with the strong hybridization between S 3p and metal d orbitals, shifts the d-band center and redistributes local charge. Because *OOH, *O, and *OH bind to the metal center through different configurations and orbital interactions, their adsorption free energies respond differently to the d-band shift. In particular, the axial S atom can serve as a hydrogen-bonding or proton-relay site to selectively stabilize *OOH, while the metal center primarily governs *OH and *O adsorption. Such a dual-site interaction decouples the adsorption of *OOH from that of *OH, thereby alleviating the traditional scaling constraint. DFT calculations on S-Fe-NC/rGO indeed show that axial S optimizes charge redistribution and enhances binding with oxygen intermediates, yielding a low ΔE of 0.64 V [110]. Similarly, in N/P-coordinated Fe sites, P introduction modifies the electronic structure of Fe, optimizing intermediate binding energies and enabling a high ORR half-wave potential of 0.88 V and a low OER overpotential of 300 mV [113]. These results suggest that asymmetric heteroatom coordination can independently tune the adsorption free energies of *OOH, *O, and *OH, providing a viable route to break the conventional ORR/OER scaling relationships. Future work combining operando spectroscopy with DFT calculations is needed to quantify deviations from scaling relations and to establish a more general design principle for asymmetric single-atom sites [114]. However, this effect depends on the specific metal center and coordination structure and needs to be further confirmed by theoretical calculations.

Chen et al. constructed a three-dimensional (3D) porous framework with abundant FeN_4_S_1_ sites by pyrolyzing a graphene oxide (GO)-coated zeolitic imidazolate framework-8 (ZIF-8) composite (Figure 10b) [110]. Owing to the modulation effect of the 3D porous framework and axial sulfur atoms, this structure could accelerate the reaction kinetics of both the oxygen reduction reaction (ORR) and the oxygen evolution reaction (OER). Density functional theory (DFT) calculations revealed that these axial S atoms can optimize electron redistribution, which facilitates surface charge polarization and improves the binding strength with oxygen intermediates. The optimal S-Fe-NC/rGO catalyst exhibited a low voltage gap (ΔE) of 0.64 V between the ORR half-wave potential and the OER potential at 10 mA cm^−2^, highlighting its excellent bifunctional electrocatalytic activity. The assembled rechargeable liquid-state zinc–air battery (ZAB) with S-Fe-NC/rGO as the air cathode delivered a peak power density of 154.05 mW cm^−2^ and outstanding durability exceeding 900 h [110].

##### N/Cl Coordinated Single-Atom Catalysts (N/Cl Coordinated SACs)

As an emerging frontier in the design of single-atom catalysts, nitrogen/chlorine co-coordinated single-atom catalysts finely regulate the charge density of the catalytic center via the extremely strong electronegativity of halogens. The axial coordination between chlorine atoms and metal single-atom sites induces a remarkable electron transfer effect, which directionally breaks the in-plane symmetry of the active center. This halogen-induced electronic regulation not only facilitates the maintenance of the single-atom dispersion state inside aerogels but also enables an optimal shift in the binding energy of oxygen-containing intermediates. In zinc–air battery applications, this property enables highly reversible oxygen electrocatalysis and guarantees excellent long-cycle cycling stability.

Bu et al. adopted NaCl and Fe(acac)_3_@ZIF-8 as precursors and successfully developed a plane chlorination engineering for Fe-N_4_ active sites via high-temperature co-pyrolysis under an inert atmosphere (Figure 10c) [111]. The Fe-N_4_/CNCl catalyst fabricated through this planar chlorination engineering strategy achieved a half-wave potential of 0.917 V vs. RHE in the electrocatalytic oxygen reduction reaction (ORR), which was 49 mV and 72 mV higher than that of the conventional Fe-N_4_/CN catalyst and commercial 20 wt% Pt/C catalyst, respectively. Furthermore, the Fe-N_4_/CNCl catalyst exhibited outstanding stability during 25,000 cycle tests. In zinc–air battery tests, the Fe-N_4_/CNCl catalyst delivered a maximum power density of 228 mW cm^−2^ and maintained excellent stability throughout 150 h of charge–discharge cycling (Table 3). Density functional theory calculations revealed that the adjacent C-Cl bond can effectively break the geometric symmetry of Fe-N_4_ active sites, downshift the d-band center of Fe, promote the reduction and desorption of OH* intermediates, and significantly reduce the energy barrier of the rate-determining step in the oxygen reduction reaction (ORR). This work demonstrated the great potential of the plane chlorination engineering strategy in completely breaking the geometric symmetry of conventional metal-N_4_ sites to substantially enhance ORR activity [111].

##### N/P Coordinated Single-Atom Catalysts (N/P Coordinated SACs)

The construction of nitrogen/phosphorus co-coordinated single-atom catalysts (SACs) by combining phosphorus with nitrogen can establish a unique local coordination microenvironment by leveraging the distinctive electron-donating property of phosphorus and its larger atomic radius. The introduction of phosphorus induces significant structural distortion and modulates the local electronic state of the central metal atom. When such asymmetric active sites are anchored inside biomass-derived carbon aerogels, the catalyst exhibits excellent electron transfer capability and tuned intermediate binding affinity, enabling extremely high peak power density for next-generation zinc–air batteries.

The AG-FeN_3_P_1_ catalyst supported on agarose-derived carbon is a typical example of nitrogen/phosphorus co-coordinated single-atom catalysts, which demonstrates that directional introduction of heteroatoms can break through the limitations of traditional FeN_4_ systems (Figure 10d) [112]. Through a multi-stage templating strategy supplemented by secondary pyrolysis treatment, the catalyst formed a unique honeycomb structure with an extremely high specific surface area (1635 m^2^ g^−1^) and a hierarchical pore structure. Extended X-ray absorption fine structure (EXAFS) analysis confirmed that it has a tetragonal planar coordination configuration of three nitrogen atoms and one phosphorus atom (coordination number: 3.0 ± 0.2 for nitrogen, 0.9 ± 0.1 for phosphorus). The introduction of phosphorus induced critical electronic perturbation, reducing the Bader charge of the iron site from +1.26 eV to +0.50 eV, while shifting the d-band center down to −0.807 eV, thereby optimizing the binding energy of reaction intermediates. This electronic regulation endowed the catalyst with excellent trifunctional catalytic activity: the half-wave potential for the oxygen reduction reaction (ORR) reached 0.90 V, and the overpotentials for the oxygen evolution reaction (OER) and hydrogen evolution reaction (HER) were 315 mV and 280 mV, respectively, outperforming the benchmark platinum on carbon (Pt/C) and ruthenium dioxide (RuO_2_) catalysts. Compared with the FeN_4_ system, this catalyst had a higher dissociation energy (ΔGdisso = 1.29 eV versus 1.23 eV for FeN_4_) and more favorable adsorption energies for O_2_ (−0.99 eV) and H_2_ (−0.22 eV), which proved that nitrogen/phosphorus co-coordination can generate a synergistic gain effect in the design of single-atom catalysts [112] (Table 3).

**Table 3:** Summary of performance parameters of the non-noble metal-doped carbon aerogels.

Strategy	E_1/2_ (V)	η (mV)	Pmax (mW cm^−2^)	Stability (h)	Ref.
N, P + FeP	0.84	-	-	160	[98]
N + FeCo	0.88	378	240	2000	[100]
N + CoFe	0.86	339	127	176	[101]
Co SAs	0.84	242	135	138	[102]
Fe/N co-doping	0.90	-	125	-	[99]
Co SAs	0.86	-	206	-	[109]
Fe SAs	0.92	-	228	150	[111]
Fe SAs	0.90	315	-	-	[112]
N + FeCo	0.81	349	-	10,000 s	[115]

Table 3 summarizes the performance parameters of non-noble metal-doped carbon aerogels used as air cathode materials for zinc–air batteries, revealing clear differences among various metal species and coordination strategies. The Fe-Co-based systems show relatively balanced performance [100], with the N + FeCo catalyst achieving an ORR half-wave potential of 0.88 V, a peak power density of 240 mW cm^−2^, and the longest cycling stability of 2000 h, whereas the Co single-atom catalyst exhibits the lowest OER overpotential of 242 mV but a lower ORR activity of 0.84 V [102]. In comparison, Fe single-atom sites provide the highest ORR half-wave potential of 0.92 V and a peak power density of 228 mW cm^−2^, but their cycling stability is limited to 150 h, indicating that higher intrinsic activity does not necessarily correspond to longer operational durability [111]. Similarly, the FeN_3_P_1_ catalyst maintains a high ORR half-wave potential of 0.90 V with an OER overpotential of 315 mV [112], while the N + FeCo system shows a lower ORR activity of 0.81 V despite its relatively low OER overpotential of 349 mV, further demonstrating the trade-off between different catalytic parameters [115]. Therefore, the data in Table 3 suggest that optimizing non-noble metal-doped carbon aerogels requires a balance among ORR/OER activity, power output, and long-term stability rather than maximizing a single performance parameter.

## 4. Application of Bio-Based Carbon Aerogels

Bio-based carbon aerogels possess three-dimensional porous frameworks, high conductivity and tailorable mechanical properties. These structural advantages enable them to realize electrocatalysis and mass transport in batteries, as well as accomplish mechanical-to-electrical signal conversion in sensors.

### 4.1. Zinc–Air Battery Applications

**Figure 11:**
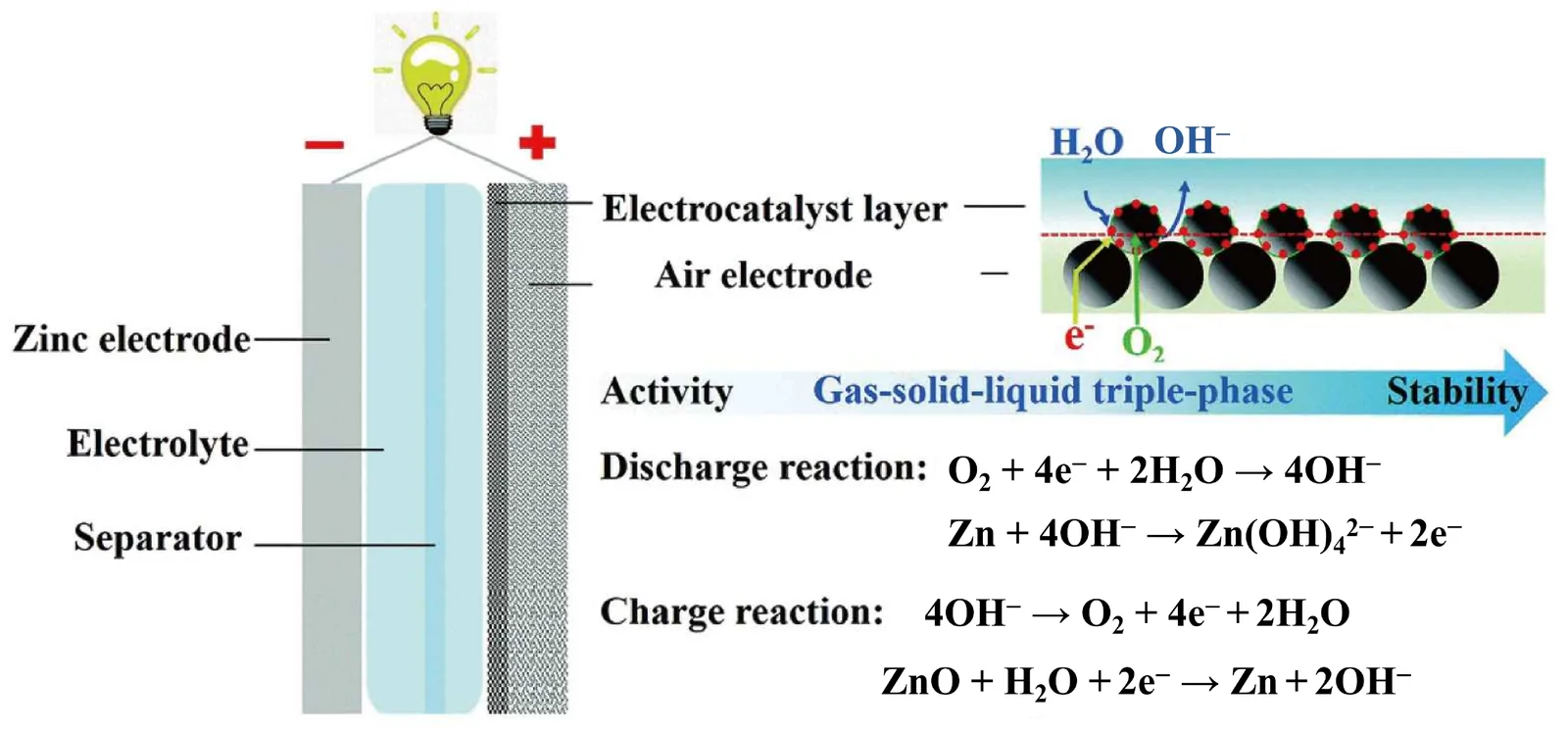
Schematic diagram of zinc–air battery [116]. Copyright 2025, Springer Nature.

A typical zinc–air battery consists of four primary components: a zinc electrode, an air electrode, an electrolyte and a separator. (Figure 11) The zinc anode is usually made of metallic zinc or its alloy, which is the negative material that provides electrons. The air cathode, where the reduction reaction of oxygen occurs, is a multi-layer structure composed of a catalytic layer, a gas diffusion layer, and a current collector. The electrolyte is commonly an alkaline solution, such as potassium hydroxide (KOH), which can facilitate the ionic conduction between the electrodes and ensure the transfer of internal charge. The separator is positioned between the positive and negative electrodes to prevent short circuits while allowing ions to pass through. The performance of each component directly influences the energy density, power density, cycle life and safety of zinc–air batteries.

The operational principle of a zinc–air battery is based on the oxidation of zinc at the anode and the reduction reaction of oxygen from air at the cathode. During discharge, the zinc anode undergoes an oxidation reaction and loses electrons to produce zinc ions, which migrate into the electrolyte [117]. These electrons flow through an external circuit to the air electrode. Simultaneously, oxygen from the air diffuses into the cathode, where it is reduced by these electrons in the presence of water to produce hydroxide ions. The zinc ions then combine with hydroxide ions in the electrolyte to form zincate species. The charging process is essentially the reverse of these reactions, where zinc is electrodeposited back onto the anode and oxygen is evolved from the cathode. The overall cell reaction equation is as follows:

(1) 2Zn↓ + O2 ⇔ 2ZnO↓

#### 4.1.1. Construction Strategies of Air Electrodes

Currently, two main approaches are employed for constructing air electrodes in Zn-air batteries. The first method is the traditional coated electrode approach, where powdered catalysts are combined with polymer binders like PTFE and conductive materials, and this mixture is then spread onto current collectors such as carbon paper, carbon cloth, or metal foam. Although this technique is well-established, the use of binders introduces several issues: increased resistance, blockage of active sites, and hindered mass transport. Moreover, during long-term operation or under mechanical deformation, the catalyst layer tends to detach from the substrate, severely compromising battery stability [118].

The second method involves self-supported monolithic electrodes. This structure eliminates the need for binders by directly immobilizing electrocatalytic active components onto a conductive substrate in situ. Such a design reduces the limitations associated with polymer binders and improves the contact between catalytic sites and conductive frameworks, which is beneficial for electron transfer and mass transport [109]. By tailoring the morphology and microstructure of self-supported electrodes, the interaction between active components and reactants can be further enhanced, promoting both mass and charge transfer, thereby improving catalytic activity and long-term stability [16].

#### 4.1.2. Self-Supporting Air Electrode

The air cathode is responsible for oxygen diffusion, electrolyte wetting, electron transport, and interfacial electrochemical reactions; hence, its structure plays a crucial role in the performance of the battery [119]. Carbon aerogels are characterized by a three-dimensional porous network that facilitates electron transport, provides open channels for oxygen diffusion, and promotes the synergistic transport of oxygen, ions, and electrons within the electrode, which mitigates polarization and enhances reaction kinetics. Moreover, the freestanding network of carbon aerogels shows remarkable structural stability during long-term electrochemical cycling [120], which is crucial for inhibiting carbon corrosion and structural degradation under continuous operation. The interconnected carbon framework can also effectively stabilize the catalytic sites, thereby reducing the risk of active material detachment during the cycling process [118]. Consequently, carbon aerogels can serve as support scaffolds for air cathodes in rechargeable zinc–air batteries.

The unique three-dimensional porous framework of carbon aerogels provides a suitable platform for constructing self-supported air electrodes. Shen et al. prepared self-supporting carbon fiber aerogels by in situ coupling Fe and N using bacterial cellulose as the precursor [109]. Deng et al. directly fabricated three-dimensional nitrogen-doped carbon self-supporting electrodes (TNCSE) from natural wood via heat treatment and acid activation [121]. The electrode preserved the hierarchical porous structure of the original wood, and its carbon skeleton contained abundant defect sites and nitrogen-doping sites.

#### 4.1.3. Flexible Zinc–Air Batteries

The rapid development of flexible wearable electronic devices has put forward higher requirements for the mechanical flexibility of energy storage devices. Conventional air cathodes are fabricated by coating a mixture of powder catalysts and binders onto current collectors. The catalytic layers are prone to cracking and peeling under repeated bending or stretching, which fails to meet the application requirements of flexible devices. The three-dimensional porous network and self-supporting properties of bio-based carbon aerogels offer an effective solution to this challenge. Their integrated structure can serve both as a carrier for catalytic active sites and as a mechanical skeleton to withstand deformation. At present, flexible zinc–air batteries mainly have three configurations: sandwich type, coplanar type, and fiber type.

##### Sandwich Type

**Figure 12:**
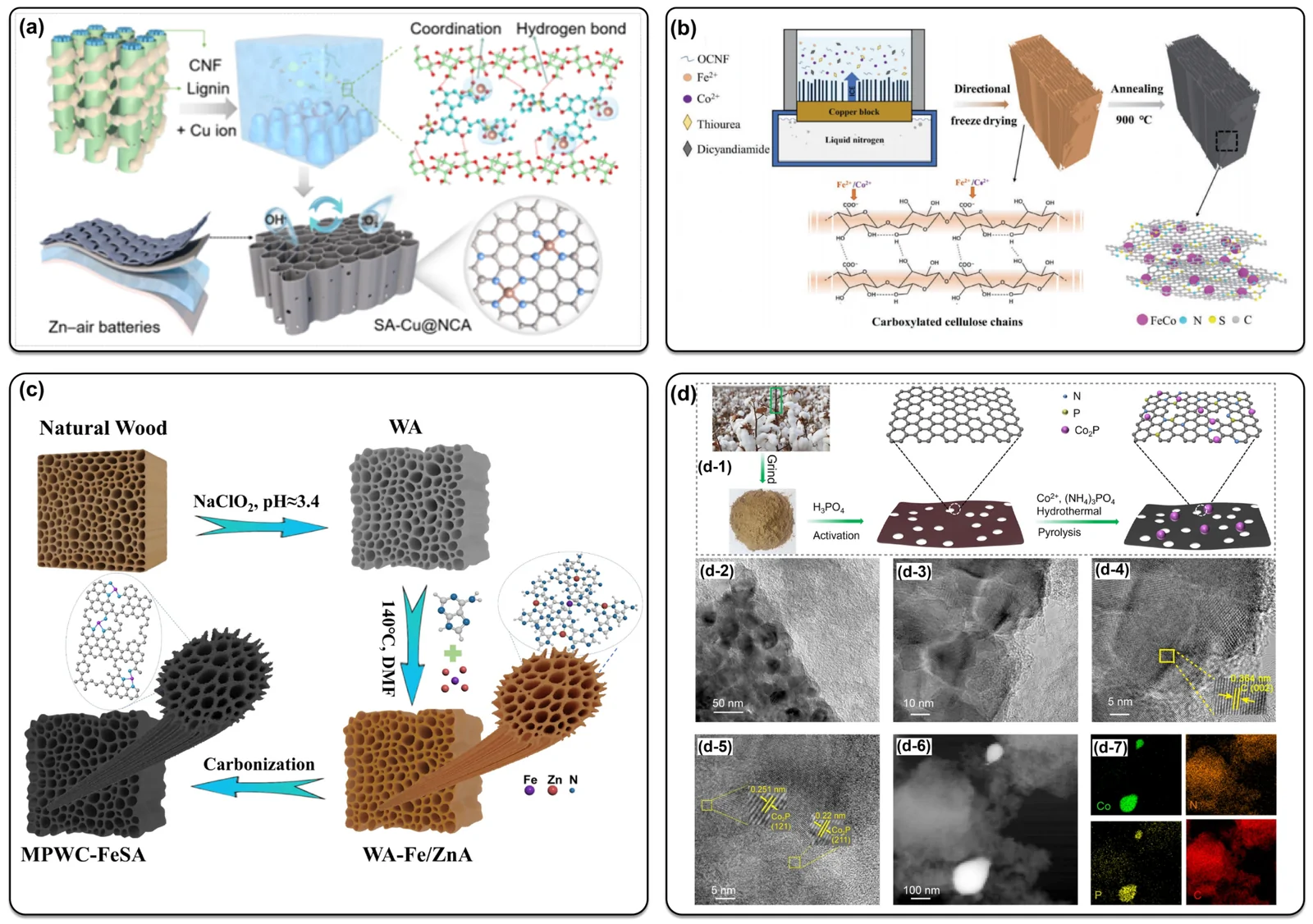
(**a**) Synthesis of SA-Cu@NCA [122]. Copyright 2025, ACS. (**b**) Schematic diagram of the synthesis process [82]. Copyright 2023, Springer Nature. (**c**) Illustration of the procedure for synthesizing MPWC-FeSA catalysts [123]. Copyright 2025, Elsevier. (**d**) Preparation route of Co2P@NPAC [124]. Copyright 2024, John Wiley and Sons.

The sandwich type is the most widely adopted structure, which is fabricated by sequentially and parallelly stacking the zinc anode, gel electrolyte, and porous air electrode layer by layer, and possesses advantages including a simple structure and large interfacial contact area. Chen et al. fabricated carbon aerogels with single-atom sites using natural wood as the precursor (Figure 12a) [122]. This material featured an ordered microstructure and M-N_4_ (M = Cu, Fe, Co) active sites, with a half-wave potential of 0.881V. The flexible zinc–air battery was assembled in a typical sandwich configuration, in which the zinc anode, gel electrolyte, and carbon aerogel air electrode were sequentially stacked layer by layer, fully exploiting the large interfacial contact area characteristic of this structure. After being assembled into flexible zinc–air batteries, the device achieved an open-circuit potential of 1.42 V, a peak power density of 98.5 mW cm^−2^, and a specific capacity of 721.9 mAh g^−1^. The battery can operate stably for 60 h under a current density of 5 mA cm^−2^, with a voltage difference of approximately 0.81 V during cycling. The charge–discharge curves remained nearly unchanged when the bending angle ranged from 0° to 150°. This battery possessed both favorable mechanical flexibility and electrochemical stability [122]. Zhang et al. prepared a honeycomb-like N,S co-doped FeCo alloy carbon aerogel (NSCA/FeCo) from cellulose nanofibers via directional casting (Figure 12b) [82]. The catalyst achieved an ORR half-wave potential of 0.85 V and an OER overpotential of only 335 mV at 10 mA cm^−2^. Following the sandwich-type architecture, the flexible ZAB was constructed by laminating the zinc foil, gel electrolyte, and NSCA/FeCo air electrode in a parallel layered arrangement, benefiting from its simple fabrication process. The assembled device delivered a maximum power density of 43.0 mW cm^−2^ and achieved a charge–discharge stability of over 450 cycles. Moreover, the battery displayed excellent flexibility under various bending conditions. Sun et al. prepared hierarchically porous wood-derived carbon aerogels loaded with atomically dispersed Fe-N_3_ sites (denoted as MPWC-FeSA) using natural wood as a precursor (Figure 12c) [123]. This catalyst exhibited excellent ORR activity in alkaline media, with an onset potential of 0.99 V and a half-wave potential of 0.85 V. In the long-term stability test, the catalyst retained 91.84% of its initial current after 50,000 s. The flexible quasi-solid-state zinc air battery (QZAB-MPWC-FeSA) adopted a sandwich structure, with the zinc anode, quasi-solid gel electrolyte, and MPWC-FeSA air cathode stacked in sequence to form an integrated layered device, ensuring stable interfacial contact under deformation. More importantly, the as-assembled battery also demonstrated outstanding electrochemical performance, achieving an open-circuit voltage of 1.42 V and a peak power density of 128.3 mW cm^−2^. When charge–discharge tests were conducted at a current density of 5 mA cm^−2^, this flexible battery can operate stably for 85 h. The charge–discharge curves show almost no significant change under bending conditions, confirming the material’s excellent flexibility. Li et al. prepared Co_2_P nanoparticles supported on nitrogen- and phosphorus-co-doped carbon carriers (Co_2_P@NPAC) using cotton stalk as the biomass precursor (Figure 12d) [124]. The catalyst exhibited excellent electrochemical activity, with an ORR half-wave potential of 0.85 V and an OER overpotential of only 261 mV at 10 mA cm^−2^. The flexible Zn-air battery was fabricated via sandwich assembly, in which the Co_2_P@NPAC air electrode, gel electrolyte, and zinc anode were superimposed layer by layer to construct a compact and mechanically robust cell configuration. The as-assembled device delivered an open-circuit voltage of 1.38 V and a peak power density of 75 mW cm^−2^. More importantly, this flexible ZAB maintains a stable charge–discharge voltage gap under various bending states, demonstrating both outstanding mechanical flexibility and electrochemical stability.

Although flexible Zn-air batteries have demonstrated retained electrochemical performance under bending, mechanical durability cannot be fully ascertained from bending tests alone. A critical comparison of the reported flexible devices shows that most studies evaluate flexibility only by bending at a fixed angle or within a certain angular range, whereas key parameters such as bending radius, number of bending cycles, twisting, folding, tensile/compressive deformation, and simultaneous electrochemical–mechanical testing are rarely reported. For example, Li et al. reported similar charge–discharge profiles for a flexible ZAB at bending angles of 45°, 90°, 135°, and 180°, and the profiles remained nearly unchanged after 1000 folding/unfolding cycles at 0.1 mA cm^−2^ [125]. Hang et al. further demonstrated a power density of 76.4 mW cm^−2^ when the ZAB was bent to 150°, compared with 114.7 mW cm^−2^ in the flat state, and the battery maintained stable operation for 500 h [126]. However, the deformation mode, bending angle or strain, number of cycles, and electrochemical testing conditions vary considerably among studies, making direct comparison of mechanical durability difficult. Therefore, repeated mechanical deformation should be evaluated together with electrochemical performance to better assess the long-term reliability of flexible ZABs.

##### Coplanar-Type

In the coplanar type, the zinc anode and air electrode are arranged side by side on the same side of the gel electrolyte, which can effectively avoid the interlayer delamination issue of stacked structures upon repeated bending. Tang et al. designed an N,S co-doped carbon-confined Co_9_S_8_ nanoparticle composite material (Co_9_S_8_@NSIC) with an inverse opal structure and employed it as a bifunctional oxygen electrocatalyst for rechargeable zinc–air batteries [127]. With a rational in-plane electrode layout, the assembled coplanar all-solid-state rechargeable zinc–air batteries (OAR-ZABs) exhibited outstanding coplanar integration capability and high flexibility. Fang et al. fabricated a coplanar flexible solid-state Zn-air battery using pollen-derived N,S co-doped porous carbon decorated with Co_9_S_8_/Fe_3_S_4_ hybrids [128]. The battery delivered an open-circuit voltage of 1.415 V, a peak power density of 78 mW cm^−2^, and a specific capacity of 785 mAh g^−1^. It maintained stable charge–discharge performance under various bending states and achieved 150 stable cycles.

##### Fiber Type

Fiber-type flexible Zn-air batteries feature a one-dimensional coaxial structure, where the zinc anode is located at the center, its surface is coated with gel electrolyte, and the air cathode is wound on the outer layer. Compared with sandwich-type and coplanar designs, this configuration offers greater flexibility and weavability. Jiang et al. constructed a flexible bifunctional electrode denoted as IrCoO_x_/CNT by growing amorphous IrCoO_x_ nanosheets in situ on carbon nanotube films [129]. At a current density of 10 mA cm^−2^, the overpotential of the oxygen evolution reaction on this electrode was only 180 mV, and the half-wave potential of the oxygen reduction reaction reached 0.86 V, and the bifunctional activity parameter ΔE was as low as 0.55 V. Based on this electrode, the researchers developed a fast-charging fiber-type Zn-air battery, which delivered a maximum charging current density of 6 mA g^−1^ and improved energy efficiency to 1.62 times that of the original device. Wang et al. employed a NaCl-assisted electrospinning strategy to prepare self-supporting carbon fibers with a solid core-mesoporous shell structure, which can be used as bifunctional oxygen electrocatalysts [130]. The assembled all-solid-state flexible zinc–air battery exhibited an open-circuit voltage of 1.39 V, a peak power density of 81.7 mW cm^−2^, and a cycling life extended to 33 h.

### 4.2. Applications of Pressure Sensors

Beyond zinc–air batteries, the structural features of biomass-derived carbon aerogels also enable their application in pressure sensing. The combination of interconnected conductive networks, porous frameworks, and mechanical flexibility allows these materials to function not only as efficient air cathodes but also as pressure-responsive sensing elements.

#### 4.2.1. The Main Principle of Flexible Pressure Sensors

Carbon aerogels are composed of an interconnected nano-scale carbon framework network, and their typical characteristics include high porosity, low density, and excellent overall structural integrity [131]. The interconnected carbon framework constructs a stable electron transport network, endowing the material with the ability to support itself, and this structural feature makes carbon aerogels significantly different from traditional carbon materials formed by the accumulation of discrete particles [16]. The interior of carbon aerogel possesses a hierarchical pore structure, which provides a structural basis for its tunability.

The material undergoes reversible deformation when subjected to external mechanical forces while still maintaining the integrity of its overall conductive network. This deformation alters pore dimensions and the contact mode of the framework, thereby regulating the distribution of conductive pathways [132]. The effective contact area between adjacent carbon skeletons also changes with the variation in stress, thereby affecting contact resistance. Moreover, during the loading and recovery process, the three-dimensional conductive network constantly reconfigures, enabling dynamic adjustments in the electron migration path. Due to the excellent elastic recovery ability of carbon aerogels, the above-mentioned structural and electrical changes are typically highly reversible and repeatable. Thus, the relationship between the structure, conductivity, and mechanical behavior is an important physical basis of carbon aerogels.

#### 4.2.2. Key Performance Parameters of Pressure Sensors

The performance of flexible pressure sensors is generally evaluated using several parameters, including sensitivity, detection range, detection limit, response/recovery time, hysteresis, repeatability, cycling stability, signal drift, frequency response, and environmental stability. Sensitivity reflects the magnitude of the electrical response induced by a given pressure change, while the detection range determines the applicable pressure regime. Response and recovery times characterize the dynamic sensing capability, whereas hysteresis and cycling stability reflect the reversibility and reliability of the sensor during repeated loading and unloading.

Beyond sensitivity, response/recovery time, strain tolerance, and cycling stability, hysteresis and signal drift are also important for assessing the reliability of pressure sensors, particularly under repeated or long-term loading. The detection limit and frequency response determine the ability to resolve weak and dynamic pressure signals, respectively, while humidity and temperature may affect both the electrical and mechanical responses of porous sensing networks. Memory effects after repeated deformation, together with device-to-device variation and baseline instability, may further influence signal accuracy and reproducibility [133]. These parameters should therefore be considered when evaluating the sensing performance and reliability of bio-based carbon aerogel pressure sensors.

#### 4.2.3. Effect of Pore Architecture on Pressure-Sensing Performance

The pore architecture of carbon aerogels has an intrinsic correlation with pressure-sensing performance. Pore size, porosity, pore connectivity, pore orientation, and the mechanical stability of pore walls collectively determine the deformation behavior of the aerogel and the evolution of its conductive network under compression. Different pore architectures can therefore produce distinct sensing characteristics.

Layered structures derived from the directional growth of lamellar ice crystals consist of stacked and undulating pore layers. Under compression, the variation in interlayer spacing and relative sliding or deformation between adjacent layers can generate pronounced changes in conductive contacts, resulting in high sensitivity, particularly in the low-pressure region. However, excessive compression may cause interlayer collapse, thereby limiting the working range and long-term stability. Jiang et al. designed a vein-like network and pillar-reinforced wavy layer structure using nanocellulose as the skeleton and alkali-treated polyimide nanofibers as interlayer struts, effectively preventing interlayer collapse during compression. This pressure sensor achieved an ultrahigh sensitivity of 59.70 kPa^−1^ in the 0–100 Pa region and retained 96.7% of its initial stress after 20,000 compression cycles [134]. In addition, Dong et al. fabricated a three-dimensional layered structure via bidirectional freezing, which exhibited a sensitivity of 25.2 kPa^−1^ in the pressure range of 0–12.5 kPa [135].

Conversely, honeycomb structures, typically derived from columnar ice crystal growth during directional freeze drying, are characterized by regularly arranged and interconnected cellular pores. During compression, this architecture efficiently distributes external stress across the cell walls, preventing localized collapse and thereby enhancing both mechanical robustness and cycling stability. However, the relatively uniform pore size may limit the deformation gradient and thus narrow the detectable pressure range. A typical example is that the micro-wrinkled honeycomb structure fabricated by Li et al. achieved a sensitivity of 48.74 kPa^−1^ yet possessed a relatively narrow effective detection range [136]. The graphene-reinforced porous network developed by Lv et al. can extend the detection range to 300 kPa, albeit with low sensitivity [137]. The above comparison reveals that honeycomb architectures are well suited for broad-range, long-durability sensing, while layered structures are more appropriate for high-precision, low-pressure detection where maximum sensitivity is the primary requirement. Quantitative studies further indicate that the sensing response is closely associated with the evolution of the porous conductive network during compression. In honeycomb aerogels, a larger pore aspect ratio and thinner pore walls facilitate structural deformation and the formation of conductive contacts, resulting in a sensitivity of up to 1.33 kPa^−1^ and a detection range of up to 59 kPa [138]. In addition, increasing the solid content of MXene/cellulose nanofiber aerogels from 5 to 20 mg mL^−1^ increased the compressive stress to 122 kPa while reducing the pore number and pore size. The resulting piezoresistive sensor exhibited a maximum sensitivity of 167.67 kPa^−1^ over the pressure range of 1.56–26.64 kPa [139]. These results indicate that pore geometry, orientation, density, and mechanical stiffness influence the evolution of conductive contacts during compression and consequently affect the pressure response of the sensor.

Overall, the pore architecture provides an important means of balancing sensitivity, working range, mechanical resilience, and cycling stability. Layered structures generally favor large deformation and high sensitivity at low pressures, whereas honeycomb-like structures provide more uniform stress distribution and improved structural stability. Therefore, rational regulation of pore size, pore connectivity, pore orientation, and pore-wall architecture is essential for tailoring the pressure-sensing performance of biomass-derived carbon aerogels.

#### 4.2.4. Applications of Biomass-Derived Carbon Aerogel Pressure Sensors

The increasing demand for lightweight, environmentally friendly, and high-performance energy storage and sensing materials has accelerated the development of wearable electronics [140]. Bio-based materials, such as cellulose [136], alginate [141], lignin [142], and their derivatives, have emerged as ideal candidates for flexible wearable sensors, owing to their wide availability, renewability, flexibility, and excellent biocompatibility [140]. Based on these merits, bio-based carbon aerogels are particularly well-suited for pressure sensing due to their unique three-dimensional porous network structure and tailorable conductivity. These carbon aerogels possess high porosity and ultralow density, and can undergo significant yet reversible compressive deformation under external pressure, resulting in highly sensitive mechanical responses. Their continuous interconnected carbon framework not only provides excellent mechanical strength and elasticity but also establishes efficient electron transmission pathways, thereby facilitating rapid signal transmission and low-power operation. Moreover, the pore structure of the aerogels can be precisely controlled by selecting the precursor and carbonization conditions, thereby optimizing their compressive resilience and the response characteristics of the conductive network.

**Figure 13:**
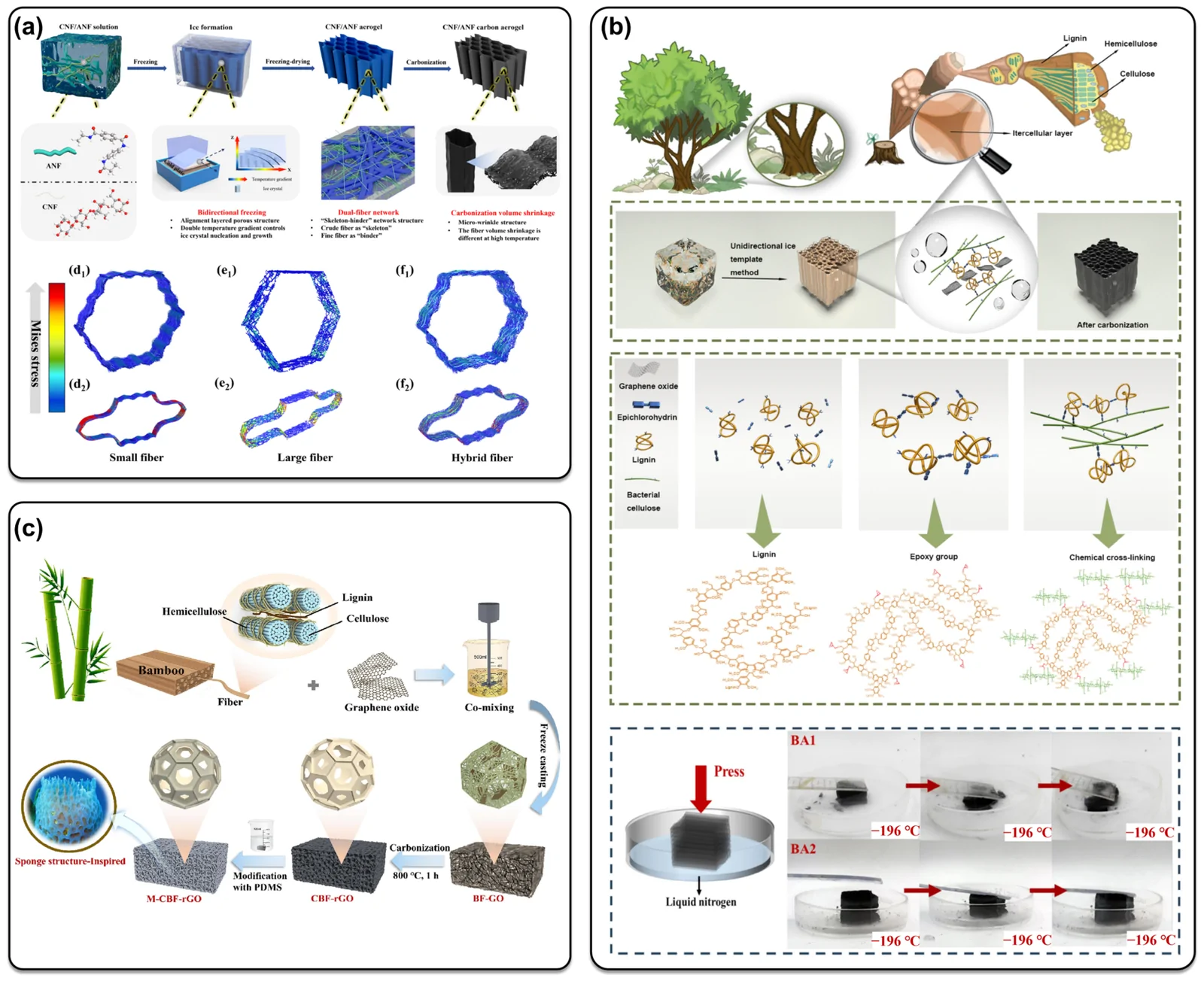
(**a**) Preparation and design concept of the CNF/ANF carbon aerogel [136]. Copyright 2025, Elsevier. (**b**) Schematic of the preparation of biomass composite aerogel [142]. Copyright 2025, Elsevier. (**c**) The design idea and synthetic process of the M-CBF-rGO aerogel flexible piezoresistive sensors [137]. Copyright 2024, Elsevier.

Li et al. prepared an ultra-lightweight carbon aerogel via co-assembly of aramid nanofibers (ANF) and nanocellulose (CNF), followed by freeze-drying and high-temperature carbonization (Figure 13a) [136]. This aerogel exhibited extremely low density, while displaying ultra-high compressibility and superelasticity. It recovered rapidly even under a 95% strain and still retained 85.49% of its initial stress after 200 compression cycles. After 10,000 cycles at a 50% strain, the performance of this aerogel showed negligible degradation, with a stress retention rate of 96.15%, demonstrating excellent fatigue resistance. Benefiting from the highly stable three-dimensional conductive network, the pressure sensor maintained stable output over a wide pressure range, achieving a sensitivity of 48.74 kPa^−1^ in the low-pressure region. The response and recovery times were 40 ms and 50 ms, respectively, highlighting structural stability and reliable signal-output capability. Inspired by the hierarchical architecture of the wood cell wall, Jiang et al. covalently crosslinked lignin with bacterial cellulose (BC) and fabricated composite carbon aerogels (BA2) with wood-like ordered porous structures via graphene oxide-induced supramolecular assembly (Figure 13b) [142]. Remarkably, the aerogel exhibited an ultralow density of 14.59 mg cm^−3^ and maintained outstanding mechanical and sensing performance even at −196 °C. After undergoing 100 compression cycles with a 50% strain, BA2 retained a high degree of 96.80% of its strength and stress of 72.66%, while its sensitivity remained as high as 2.37 kPa^−1^, enabling it to reliably detect motion and voice signals under harsh conditions.

In addition, Lv et al. prepared a superelastic spongy aerogel (M-CBF-rGO) via freeze-drying using carbonized bamboo fiber (BF) and graphene oxide (GO) as precursors, which was subsequently coated with polydimethylsiloxane (PDMS) for piezoresistive sensing applications (Figure 13c) [137]. The architecture of this material was characterized by a macroscopic cellular network woven from bamboo fibers and reinforced dense pore walls embedded with graphene oxide (GO), which endowed it with outstanding mechanical properties and sensing capabilities. In terms of mechanical properties, it could withstand up to 90% compressive strain and remained highly stable after 2500 cycles at 70% strain. For the sensing performance, its pressure detection range was as wide as 0–300 kPa, with a sensitivity of 0.59 kPa^−1^ in the low-pressure section (0–70 kPa) and 0.31 kPa^−1^ in the high-pressure section (70–300 kPa). At 70% strain, response time and recovery time were 100 ms and 20 ms, respectively. The sensor can accurately detect various physiological signals ranging from micro-frowning to joint bending.

These studies demonstrate that biomass-derived carbon aerogels can be employed for a wide range of pressure-sensing applications, including physiological monitoring, joint-motion detection, voice recognition, and other wearable sensing systems. Their sensing performance is strongly dependent on the coupling between pore architecture, mechanical deformation, and conductive-network evolution. Therefore, structural regulation provides an effective strategy for adapting biomass-derived carbon aerogels to different pressure-sensing requirements.

## 5. Conclusions

Bio-based carbon aerogels feature three-dimensional porous structures, high specific surface areas, and tunable structures, and have been extensively investigated as air-electrode materials for zinc–air batteries. Carbonization treatment following directional freeze-drying endows the material with a porous and robust carbon skeleton, and this unique structure provides an ideal support for subsequent heteroatom doping and the introduction of non-noble metals. This is of critical significance for regulating the electronic structure, constructing defect sites, and ultimately improving the bifunctional electrocatalytic activity of the oxygen reduction reaction (ORR) and oxygen evolution reaction (OER). Furthermore, this class of aerogels inherently possesses mechanical properties, including high resilience and sensing responsiveness, enabling their application in the field of pressure sensing. This dual functionality underscores their significant potential in the development of green, renewable flexible electronic devices. Compared with published reviews, the novelty of this review can be illustrated as follows. (I) The structural advantages of bio-based precursors are innovatively integrated with both electrochemical energy storage and mechanical sensing, shifting the focus from conventional carbon materials and traditional precursors to the targeted design of biomass-derived carbon aerogels and flexible device integration. (II) In response to the intrinsic heterogeneity of biomass precursors, this review systematically consolidates bottom-up strategies for the precise construction of hierarchical pore structures, and elucidates the common regulatory mechanisms underlying their three-dimensional interconnected networks, high specific surface areas, and high mechanical resilience in oxygen electrocatalysis for zinc–air batteries and highly sensitive pressure sensing.

Despite these significant advancements, there are still several challenges that remain to be addressed to unlock the full potential of bio-based carbon aerogels.

(1) Conventional single-atom catalytic sites suffer from the sluggish kinetics of oxygen electrocatalytic reactions due to their symmetric electronic structures. Future research should focus on constructing asymmetric single-atom sites co-coordinated by multiple heteroatoms (such as nitrogen/sulfur, nitrogen/phosphorus, or nitrogen/chlorine configurations) that break geometric symmetry, dynamically regulate the local electronic states, and reduce the reaction energy barrier. The catalytic activity and stability of these sites should be quantitatively correlated with their coordination environments and electronic structures.

(2) Under severe conditions such as continuous mechanical deformation and sub-zero temperatures, existing pore structures are prone to interlayer collapse and performance degradation. Optimizing hierarchical pore structures into biomimetic layered or honeycomb skeletons can enhance the structural deformation resistance and ensure stable electrochemical performance under extreme environments. During the charge–discharge process of zinc–air batteries, metallic active substances in carbon aerogels are prone to dissolution, which is particularly pronounced under high current density conditions and consequently leads to decreased stability of zinc–air batteries. Therefore, structural reinforcement and active-site protection strategies should be developed and evaluated under well-defined mechanical, temperature, and electrochemical conditions.

(3) Traditional fabrication methods are highly dependent on costly freeze-drying equipment and corrosive external chemical activators. Meanwhile, such processes are characterized by complex procedures and high energy consumption. To enable large-scale fabrication of biomass-based electrocatalysts, green, practical, and low-energy preparation methods are urgently required. For instance, the adoption of scalable atmospheric drying strategies, along with the utilization of the inherent self-activation property of industrial by-products such as black liquor, can eliminate the requirement for hazardous reagents and reduce manufacturing costs. Future studies should further quantify production yield, energy consumption, cost, and environmental impact to assess scalability.

(4) It is challenging for monolithic carbon aerogels to realize the complex structural design required for the collaborative integration of energy storage and sensing components in micro wearable devices. Therefore, there is an urgent need to develop integrated structures such as coplanar or fibrous structures that facilitate the construction of fully integrated self-powered systems in which zinc–air batteries can directly power flexible piezoresistive sensors. Beyond structural integration, future studies should also evaluate practical sensing reliability, including hysteresis, signal drift, temperature and humidity cross-sensitivity, frequency response, and device-to-device variation, under standardized testing conditions.

Future studies should place greater emphasis on quantitative evaluation of the scalability and practical reliability of biomass-derived carbon aerogels. In addition to electrochemical performance, production yield, energy consumption per unit mass, precursor variability, catalyst loading, device lifetime, and device-to-device reproducibility should be considered when assessing the transition from laboratory-scale preparation to larger-scale production. Production cost and environmental footprint should also be evaluated and compared with those of conventional carbon-based electrocatalysts under comparable performance requirements. Establishing consistent quantitative criteria for these parameters will be important for assessing the practical feasibility of biomass-derived carbon aerogels beyond laboratory-scale demonstrations.

## Data Availability

No new data were created or analyzed in this study. Data sharing is not applicable to this article.
